# The Impact of Artificial Intelligence on Facial Aesthetic Surgery: A Systematic Review

**DOI:** 10.1093/asjof/ojag114

**Published:** 2026-06-23

**Authors:** Aurelia-Alexandra Colceriu, Petru-Iulian Ifteni, Liliana Rogozea, Florin-Gabriel Leașu, David Stuckler

## Abstract

Artificial intelligence (AI) has transformed medical practice, with early adopters using custom models in imaging-heavy fields. Millions of cosmetic surgeries prompt surgeons to seek better tools amid pressure for ideal outcomes. Past reviews have covered AI in facial cosmetic surgery for both reconstructive and aesthetic purposes. There remains a need to cover AI applications in a wider range of specialties while assessing clinical relevance, readiness, and, when available, comparison with standard care. This research examines AI's impact on facial cosmetic surgery by reviewing patient care stages, surgery types, AI architectures, and model sourcing. Following PRISMA 2020 guidelines and PROSPERO registration, the authors searched PubMed and Web of Science on August 25, 2025, for English studies on facial aesthetic surgery. They included studies using AI across all care stages, with at least 4 patients. Two reviewers independently screened, assessed, and extracted data. Of 1832 records, 29 met criteria. The authors used the Cochrane ROBINS-I and RoB 2 tools for risk of bias assessment. Most studies, mainly in Asia or the United States, focus on AI tool validation, development, and applicability, with few interventional studies. Most AI models use convolutional neural networks and are commercially available. In facial aesthetic surgery, AI aids treatment planning and outcome assessment, especially in facelifts, rhinoplasty, and blepharoplasty. The results highlight AI's potential and emphasize the need for standardized reporting, updated regulations, and education programs that promote collaboration where surgeons co-design AI models.

**Level of Evidence: 4 (Therapeutic)**  
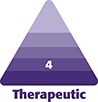

The emergence of big data and deep learning has led to significant advances in image analysis and natural language processing, with transformative implications for cosmetic medicine. Although there is clearly significant potential, debate remains among cosmetic surgeons about whether and how best AI can be integrated into clinical practice and workflows. AI is a branch of computer science that enables systems to perform complex tasks that typically require human intelligence. This enables them to make predictions, recommendations, or decisions, with or without human oversight.^1^

Multiple potential benefits exist, such as time efficiency, improved diagnostic accuracy, or predictive modeling.^2^ However, concerns arise about potential ethical or regulatory hurdles.^3^ Despite the millions of cosmetic surgeries performed worldwide, these procedures pose a unique challenge for surgeons to meet ideal beauty standards.^4^ Consequently, AI applications that enhance patient care and surgical outcomes can be highly anticipated; yet, it is not clear which of these have the potential not only to be incorporated into practice but also to yield their purported benefits for providers and patients alike.

To date, a series of systematic reviews by Stephanian et al, Espinosa Reyes et al, or Souza et al have focused on the impact of AI in facial procedures performed for both cosmetic and reconstructive reasons.^5-7^ However, as a result, these studies have only captured a limited portion of cosmetic surgeries that are adopting AI technologies. There remains a need to cover applications in a wider range of specialties, including plastic surgery, maxillofacial, ENT, ophthalmology, and dermatology, while assessing clinical relevance, readiness, and, when available, comparison with standard care. Because real-world applications increase exponentially, there is a need to map where AI is being implemented both across fields and in stages of patient care, from assessment through to evaluating outcomes.

Here to plug this gap, we performed, to our knowledge, the first comprehensive systematic review of real-world AI applications in facial aesthetic surgery. We further mapped these applications by stage of patient care, type of surgery, AI architectures, model sourcing, and whether they compared AI with clinician performance or were feasibility studies. A meta-analysis was not feasible because of the diversity of study designs, incompatible outcome measurements, and significant heterogeneity. Consequently, we chose a systematic review instead of other review formats to deliver a thorough, transparent, and reproducible summary of the latest literature, aiming to produce valuable insights for guiding future research and policymaking.

## METHODS

We performed a systematic review following the best-practice PRISMA methods using the protocol published in PROSPERO CRD420251162215, accepted on October 24, 2025.^8,9^ To reduce risks related to retrospective approval, the eligibility criteria, outcomes of interest, and synthesis plan were finalized and fully uploaded before any data extraction. No post hoc changes to criteria or outcomes were made after screening began.

### Search Strategy

We searched PubMed and Web of Science on August 25, 2025, for peer-reviewed articles on artificial intelligence (AI) and cosmetic surgery of the head and neck. To capture the diversity of both aesthetic surgeries and AI subfields, we operationalized different permutations of each keyword, leading to a conceptual framework based on a building-block approach.^10^

The key term “cosmetic surgery” was defined using principles of topographic anatomy, whereas “artificial intelligence” was examined through various subdomains. To enhance our selection of key terms, we reviewed previous systematic reviews on both major topics combined, as well as each topic individually.^11-13^ The search queries were optimized by using equivalent functions between databases. In each database, we employed advanced search functions, using Boolean operators such as “OR” and “AND” along with truncation. Additionally, we applied the “Title and abstract” field in PubMed, whereas in Web of Science, we selected the “Topic” field. The full details of the search are available in the Appendix.

### Inclusion and Exclusion Criteria

A set of inclusion and exclusion criteria was applied. Articles were included if they (1) were written in English, with an available abstract or full text; (2) were original articles published in peer-reviewed journals; (3) explicitly stated the type of AI model used and utilized its capabilities for clinical purposes (such as screening, patient assessment, treatment planning, therapeutic applications, outcome evaluation, or predictive modeling); (4) involved adults, minors with tutor consent, or patient-derived data exclusively from medical practices (eg, electronic medical records); (5) involved patients who underwent facial aesthetic surgery either before or as part of the study design; and (6) were designed as descriptive, analytical, or hybrid studies.

Studies were excluded if (1) they employed AI technologies outside surgery-related, healthcare professional use, such as patient education outside clinical encounters, workflow management, and optimization; (2) surgeries were performed for reconstructive or mainly functional reasons; and (3) studies involved case reports of 1 patient, cadaver studies, and in vitro research or were exclusively based on animal testing.

### Extraction and Analysis

Two reviewers (A.-A.C. and F.-G.L.) independently extracted data using Excel (Microsoft Corporation, Redmond, WA), and the results were then compared.^14^ The other 3 authors (L.R., P.-I.I., and D.S.) conducted a final review. Discrepancies were resolved through discussion and consensus. The following data were collected: author(s), year, sample size, study design, type of cosmetic surgery, AI architecture, type of AI access, AI tasks, clinical stage at which AI was used, measured outcomes, assessment methods, and key findings.

We performed a narrative synthesis centered on AI applications in facial cosmetic surgery because the considerable variability in outcome measures made meta-analysis infeasible. Our goal was to identify key information and patterns, later categorized by patient care stage, type of surgery, and AI technical details.

The studies were classified using an AI-focused developmental framework to highlight the primary objective and translational phase of each application. Feasibility studies assessed clinical applicability without formal validation; technical development studies focused on model design or optimization; technical validation studies evaluated performance against a reference standard; and comparative studies directly compared AI-based applications with standard care.

We classified the AI model sourcing based on system accessibility and developer control. Commercially available AI refers to licensed tools, accessible to external users. Open-source AI refers to models that function based on publicly available code. Proprietary AI refers to custom-developed systems with restricted access. Hybrid AI systems combine open-source architectures or algorithms with proprietary elements like private datasets, commercial software, or unpublished code. They are partly transparent, with restricted access to training data, source code, or deployment pipelines, and often limited external validation.

The primary outcomes were categorized by surgical procedure (eg, blepharoplasty, facelift, and rhinoplasty) and differentiated by the clinical stage of AI use. To identify various patterns that can emerge from specific AI models, the analysis has also focused on their architecture, access types, and, when available, comparison to standard care.

### Quality Assessment

The quality of all included studies was assessed using the Cochrane ROBINS-I tool for nonrandomized clinical studies and the RoB 2 tool for randomized clinical studies.^15,16^ Although these tools differ, common shared risk of bias domains include those assessing intervention, missing data, outcome measurement, or selection reporting. Two authors (L.R. and P.-I.I.) independently evaluated the risk of bias in each study. Any discrepancies were resolved through discussion with a third author (D.S.).

## RESULTS

**Figure 1:**
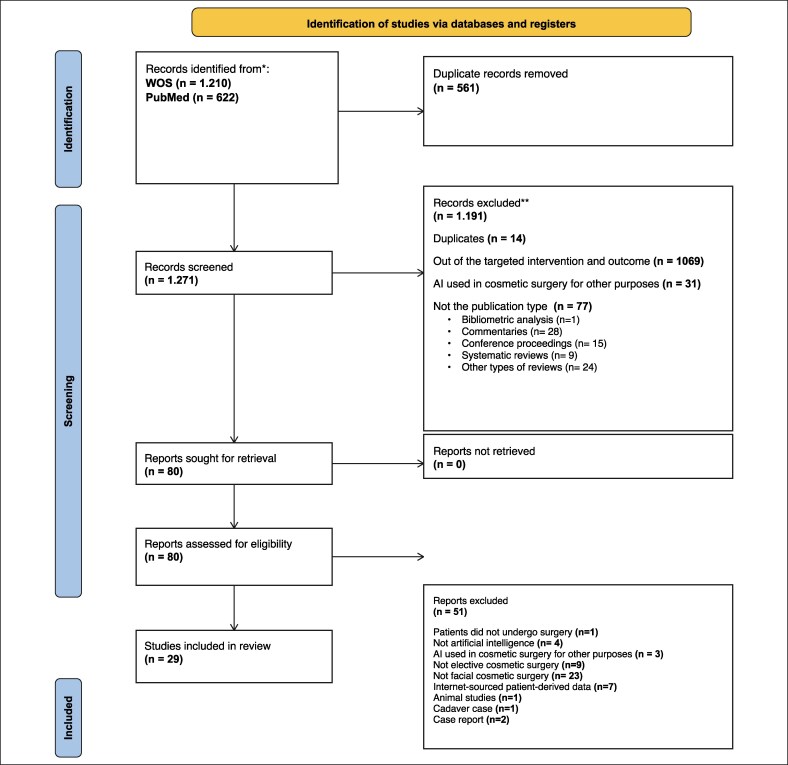
PRISMA flow diagram. WOS, Web of Science.

Our initial search identified 1210 articles in Web of Science and 622 in PubMed, which were imported into Zotero reference management software.^17^ A total of 561 articles were identified as duplicates, leaving a total of 1271 articles for screening and eligibility stages. We further excluded 1069 articles that did not meet our intervention and outcome eligibility criteria and another 31 studies that used AI in cosmetic surgery for purposes other than clinical applications. Additionally, 14 duplicates were excluded, along with 77 articles that were outside the desired category of original, primary literature. Upon full-text review of the remaining 80 articles, 51 articles were excluded for reasons related to patient data sourcing, nonelective or nonfacial aesthetic surgery, lack of AI use, or article type (animal study, case report, or cadaver study). This left a total of 29 articles for the final review, as seen in Figure 1.

Screening proceeded in 2 stages, by the authors A.-A.C and F.-G.L. Stage 1 involved independent title and screening, whereas Stage 2 comprised independent full-text review. There were no disagreements on the final set of 29 included studies.

### Overall Study Characteristics

Our systematic review encompasses 29 studies, with detailed characteristics in Supplemental Tables 1-8. Geographically, most research originated from Asian countries, followed by the United States. Most single-center studies originate from the United States (*n* = 9), followed by Asian countries such as China (*n* = 7), Korea (*n* = 4), Taiwan (*n* = 1), and Iran (*n* = 1). Additional single-center studies come from Turkey (*n* = 3) and Tunisia (*n* = 1). We also included 3 multicenter studies. We observe an exponential rise in research interest, as evidenced by the annual growth in publications on AI and facial aesthetic surgery since the first publication appeared in 2019.

**Figure 2:**
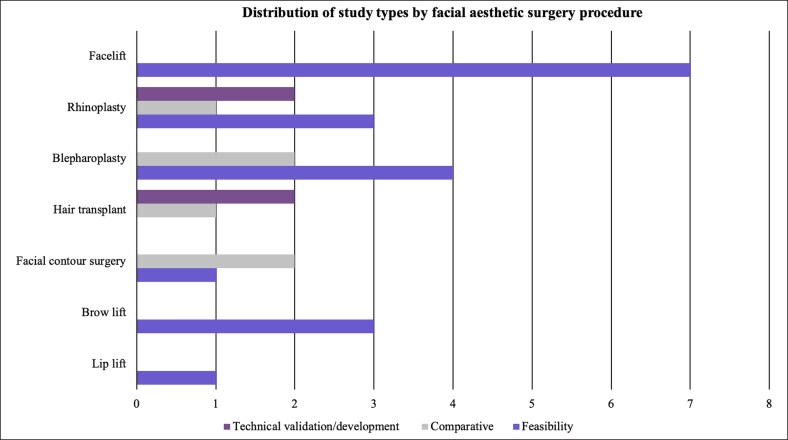
Distribution of study types by facial aesthetic surgery procedure.

We identified 3 types of studies: feasibility studies (*n* = 19) encompassing 2512 patients that evaluated AI's practicality; technical validation or development studies (*n* = 4) with a total of 355 patients that tested whether AI could perform in a real-world environment, and comparative studies (*n* = 6) with 210 patients that examined AI's potential against standard practice. The distribution of study types by facial aesthetic surgery procedures is shown in Figure 2.

**Figure 3:**
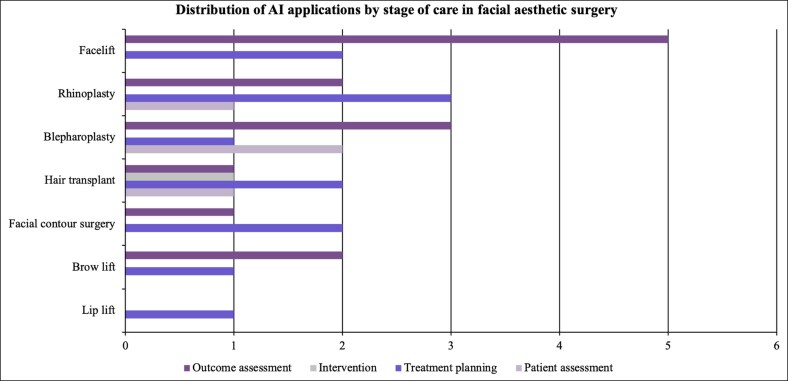
Distribution of artificial intelligence (AI) applications by stage of care in facial aesthetic surgery.

**Figure 4:**
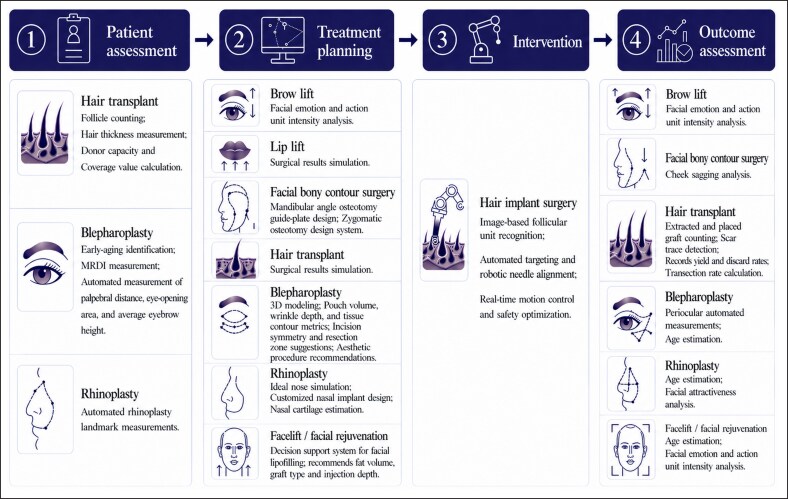
Distribution of artificial intelligence (AI) applications in facial aesthetic surgery by stages of care. MRD1, margin reflex distance 1; 3D, 3-dimensional. Generative AI (ChatGPT 5.4, OpenAI, San Francisco, CA) was used to create the image. The authors reviewed, edited, and take full responsibility for the content.

As seen in Figure 3, we also uncovered multiple applications of AI across 4 phases of cosmetic surgery patient care: patient assessment (*n* = 4) in a total of 677 participants, treatment planning (*n* = 9) in 880 participants, intervention (*n* = 1) in 13 patients, and outcome assessment (*n* = 17) in a total of 1507 participants. These applications were distributed across diverse facial cosmetic surgeries, including facelift (*n* = 7), rhinoplasty (*n* = 6), blepharoplasty (*n* = 6), browlift (*n* = 3), hair transplant (*n* = 3), facial contouring (*n* = 3), and lip lift (*n* = 1). Figure 4 illustrates how AI applications are distributed across different care stages and procedure types.

### Lip Lift Applications (*n* = 1)

As shown in Supplemental Table 2, we found only 1 study where AI was employed to facilitate treatment planning of lip lift surgery. Huang et al assessed the feasibility of a commercially available, generative text-to-image transformer AI model, DALL·E 2 (OpenAI, San Francisco, CA) to simulate lip lift outcomes in 4 patients.^18,19^ The authors noted that the difference between AI-generated images and actual surgical results was subtle (∼1.2 mm) and that prompting impacted the image output. Although the difference between AI forecast images and actual results was modest, the authors suggested that AI use in treatment planning could lead patients to have unrealistic expectations.

### Brow Lift Applications (*n* = 3)

We identified examples of AI being applied to brow lift surgery during 2 stages of care: treatment planning and outcome assessment (see Supplemental Table 3). All 3 brow lift studies were feasibility studies, employing the same commercially available convolutional neural network (CNN) model, FaceReader (Noldus Information Technology, Wageningen, the Netherlands), which analyzes the intensity of cardinal emotions and facial action units.^20^

In treatment planning, Zhu et al integrated AI with other simulation tools in a study involving 53 postsurgical patients.^21^ They edited photographs to vary brow rotations, using AI to measure how these rotations affected facial emotions and facial action units. The authors suggested that AI assessment of facial emotions and action units could improve patient–doctor communication and help patients anticipate the impact of surgery on their facial emotional expressions, thereby facilitating patient decision making.

In outcome assessment, both Boonipat et al and Hebel et al studied patients who underwent brow lift surgery, with 52 and 59 cases, respectively.^22,23^ The studies followed a similar methodology: the FaceReader (Noldus Information Technology) AI model was used to measure changes in expression and facial action units following cosmetic surgery.^20^ In both studies, the AI model detected statistically significant decreases in anger, increases in happiness, and reductions in brow-lowering facial action units after surgery. Hebel et al further examined how 2 different brow lift techniques could influence AI interpretations and found that the model detected more significant cardinal emotion and facial action unit changes using the standard technique, which uses the incision placement in the hair-bearing skin of the scalp, instead of the novel horizontal rhytid line placement.^23^

### Facial Bony Contour Surgery Applications (*n* = 3)

We identified 3 AI applications in facial contour surgery covering 2 stages of care: treatment planning and outcome assessment (see Supplemental Table 4).

In treatment planning, applications include the design of mandibular angle ostectomy guide plates and zygomatic osteotomy systems. Yan et al developed a custom AI model for the digital design of mandibular angle ostectomy guide plates and compared it with manual surgeons’ designs in 50 patients.^24^ Findings indicate that the AI increased safety rates (96% vs 52%) and reduced design time (average output in 25 s), with no significant differences in symmetry, plate shape, match accuracy, or aesthetic angle ratios.

Qiu et al developed a hybrid AI model for handling 3-dimensional (3D) image data to assist in zygomatic osteotomy design.^25^ They tested it against manual surgeon designs in a small, early-phase clinical trial involving 6 patients. The AI-generated bone cutting plan achieved noninferior safety outcomes, with improved symmetry and aesthetic results.

In outcome assessment, Park et al evaluated the feasibility of using AI to quantify soft-tissue ptosis following zygoma reduction surgery in 72 patients.^26^ The AI tool effectively demonstrated that no cheek sagging had occurred after surgery, through a combined analysis of facial sagging indices, cheek curvature, nasolabial, and marionette line assessment.

### Hair-Implant Applications (*n* = 3)

As shown in Supplemental Table 5, hair-implant surgery is the only type of surgery where we found AI applications across all stages of care.

With regard to the patient assessment phase, the technical development and validation study conducted by Erdogan et al analyzed the scalp images and hair strands from 47 preoperative and postoperative patients.^27^ Authors employed an AI-powered robotic system (KEBOT) to quantify hair density, follicle count, and thickness, thereby informing donor scalp capacity. The AI model demonstrated technical accuracy, consistent results, and error rates under 5% when tested against manual nurse labeling or scanning electron microscopy.

In treatment planning, we identified 2 studies that used AI for outcome prediction and donor scalp evaluation. Hwang et al technically developed and validated a custom, generative adversarial network-based model to predict surgical outcomes using a dataset of 1768 prehair and 1064 posthair transplantation images.^28^ The study's findings demonstrated superior region-of-interest detection compared with baseline models. In the second study by Zhu et al, an AI-assisted hair-implant robot, ARTAS (Venus Concept, Toronto, Canada), was compared with manual follicular unit extraction (FUE) in a randomized, split-scalp study of 13 patients.^29,30^ Tasks such as donor scalp assessment, follicular unit identification, and hair angle analysis augmented the robot's operational capacity during the intervention.

In the intervention phase, the same study by Zhu et al further demonstrated that the AI-enabled hair-implant robot, ARTAS (Venus Concept), was noninferior to the surgeon's manual FUE.^29,30^ The authors revealed no significant differences in yield rates and lower transection rates in the robot. Although manual FUE was more efficient overall in terms of total discard rates, the AI-based hair restoration robot showed better results for 1-hair FUE. Clinically, follicles harvested with the AI-powered robotic hair system were more likely to be of higher quality than those from FUE, with comparable safety and satisfaction rates.

In outcome assessment, the aforementioned study by Erdogan et al further showed that, besides the AI applications in the patient assessment stage, the model was able to trace scars and detect the extracted and placed grafts, with error levels below 5%.^27^

### Blepharoplasty Applications (*n* = 6)

We identified multiple AI applications through 3 stages of care: patient assessment, treatment planning, and outcome evaluation (see Supplemental Table 6).

In patient assessment, 2 studies highlight AI applications in blepharoplasty, including home-based detection of early signs of aging and automated measurement of the marginal reflex distance 1. Lian et al developed a smartphone app based on a hybrid, hierarchical attention transformer AI model called HATrans, and further tested its feasibility in a group of 454 patients.^31^ The model was designed to help identify periorbital aging signs and provide accessible, timely treatment suggestions. The authors showed that the model's recommendations were highly accepted by surgeons, with acceptance rates ranging from 89.5% to 94%. In the second study, Song et al introduced a deep learning algorithm based on an open-sourced framework to automatically measure the marginal reflex distance (a key eyelid ptosis metric) in a group of 77 patients and compared it with manual and computer-based methods.^32^ The measurement precision of the proposed tool was highly correlated with the computer-based method set as the gold standard and outperformed manual surgeons’ assessment, which was more labor intensive and error prone.

In treatment planning, Qu et al conducted a comparative study of 64 patients to assess whether their proprietary, CNN-based AI tool would affect surgeons’ performance compared with experience alone.^33^ The multichannel CNN provided 3D models, estimated pouch volume, measured wrinkle depth, and assessed tissue contour metrics for surgical planning. Authors showed that the additional use of the AI tool led to significant improvements in pouch degrees, lower eyelid wrinkles, skin gloss, and aesthetic scores. Furthermore, it reduced complication rates (from 28% to 13%) and outperformed traditional CNNs in efficiency.

In the outcome evaluation of blepharoplasty, we identified 2 AI applications: age estimation and automatic eye measurements. Chiou et al developed a software (FaceAge) comprising 4 commercially available CNNs and tested its applicability in a group of 150 patients.^34^ The researchers reported that, according to the AI outputs, blepharoplasty had a rejuvenating effect, especially in men and in those undergoing a combined approach. Kreh et al followed a similar methodology in 153 patients undergoing periorbital rejuvenation, using 4 commercial AI tools: Face++ Megvii (Beijing, China), Betaface (Munich, Germany), Facelytics (Wassa, France), and Everypixel (Everypixel Media Innovation Group, Singapore).^35-39^ Consistent with Chiou et al's findings, AI outputs showed a rejuvenation effect of periorbital surgery. Additionally, Face++ Megvii, the AI model utilized in both studies, was found to be the most accurate in age estimation.^36^

Additionally, Şimşek and Sirolu evaluated the applicability of a custom-configured open-source AI toolkit for automatically measuring 3 eye distances: palpebral distance, eye-opening area, and average eyebrow height.^40^ In a group of 55 patients, the authors demonstrated that AI measurements objectively quantified differences among blepharoplasty techniques, thereby providing a standardized, objective, and repeatable method.

### Rhinoplasty Applications (*n* = 6)

We found AI applications in rhinoplasty across 3 phases of care—patient assessment, treatment planning, and outcome assessment, as further presented in Supplemental Table 7.

In patient assessment, AI-based applications include facial anthropometry analysis. Jafargholkhanloo et al used a hybrid, cascade regression-based model to measure 11 anatomical landmarks and 9 angular parameters in a technical validation study of 100 patients.^41^ Using a comparative approach, the authors found no significant differences between AI-based and clinician manual measurements. Additionally, the AI approach provided faster results with a lower chance of error.

In the treatment planning stage, we identified 3 main areas of AI use: 3D modeling, ideal nose simulation, and custom nasal implant development. In a group of 209 patients, Li et al developed and tested an open-source deep neural network (FoldingNet) designed to reconstruct 3D noses from images and generate an ideal nose.^42^ Authors demonstrated that outputs occurred nearly in real time after data import and produced results comparable to manual, surgeon-designed ones that were set as standard. Suh et al tested the feasibility of a commercial software, Materialize Mimics (Materialise NV, Leuven, Belgium) integration with proprietary AI modules in a case series of 4 patients.^43,44^ The AI model facilitated customized nasal implant development by nasal cartilage prediction and 3D model generation. Findings showed that the combined deep learning and modeling pipeline achieved a prediction error of <1 mm when comparing simulated outcomes with actual postoperative results.

In outcome assessment of rhinoplasty, AI applications include age estimation and facial attractiveness evaluation. We included 3 feasibility studies that employed commercially available, CNN-based AI tools. Yalçın et al used Microsoft Azure Face (Redmond, WA) to assess photographs of 244 patients.^45,46^ Despite the elapsed follow-up time, AI evaluations mainly remained unchanged, which led the authors to present data as a possible antiaging effect. Khetpal et al used Haystack AI (New York, NY) in a retrospective group of 124 patients.^47^ The authors showed that AI-driven analysis interpreted rhinoplasty as a surgical procedure with rejuvenation effects and gains in attractiveness. Dorfman et al employed Microsoft Azure Face^46^ in a group of 100 patients; their findings align with previous studies: AI interpretations label postrhinoplasty patients as younger after surgery, with a mean of −3.1 years.^48^

### Facelift Surgery Applications (*n* = 7)

As shown in Supplemental Table 8, we identified AI applications for facelift surgery covering 2 stages of care: treatment planning and outcome evaluation. In treatment planning, AI facelift applications are used for predictive modeling purposes. Based on a sample size of 400 patients, Tiryaki et al prospectively validated a custom-designed AI tool.^49^ Authors acknowledged that the model could hold promise for aiding practitioners in handling patients outside the facial gold ratio, with recommendations on fat volume selection, graft type, or injection depth.

In the outcome assessment of facelifts, we included 6 studies; 5 were feasibility studies, and 1 was a comparative study that employed AI for age estimation, facial emotion intensity detection, and facial action unit detection. Regarding age estimation capabilities, 3 out of 5 studies used the same set of commercially available AI models: Face++ Megvii, Amazon Rekognition (Seattle, WA), Microsoft Azure Face, and IBM (Armonk, NY).^36,46,50,51^

In a group of 50 patients, Zhang et al found that high FACE-Q scores correlated with the AI-estimated age reduction, although patients tended to perceive themselves as significantly younger than the AI estimates.^52^ Gibstein et al analyzed a larger cohort of 105 patients and further employed AI to evaluate the impact of different facelift techniques on the rejuvenation effect, demonstrating that the skin-only approach was less effective than SMAS plication or SMAS-ectomy alternatives.^53^ Consistent with previous findings, Bouguila et al demonstrated that AI interpretations of 37 facelift patients led to a significantly younger appearance (mean, 5.57 years reduction).^54^

Another commercially available AI model, FaceX (Bangladesh and India), was used in a group of 226 patients. Elliot et al demonstrated that the AI scored high in age detection accuracy, although it tended to overestimate age.^55^ Additionally, researchers showed that no single ancillary procedure or technique provided more benefit than others. A comparative study in 48 patients by Du et al used an open-sourced, transformer-based AI (MiVOLO) and aimed for a nuanced comparison: machine versus naive observers.^56,57^ Results demonstrated that AI outperformed observers in the preoperative evaluation with fewer mean absolute errors. When AI age estimation was compared with patients’ actual age, those who appeared older than their preoperative age statistically matched clinical observations.

Turning to emotion and action unit intensity evaluation, one study by Hebel et al used FaceReader (Noldus Information Technology) and aimed to compare the effect of different surgical techniques.^20,58^ Their research involved 32 patients and revealed that AI interpreted significant improvements solely in the High-SMAS technique group, which showed increased happiness, decreased anger, and fewer negative facial action units.

### Risk of Bias

**Figure 5:**
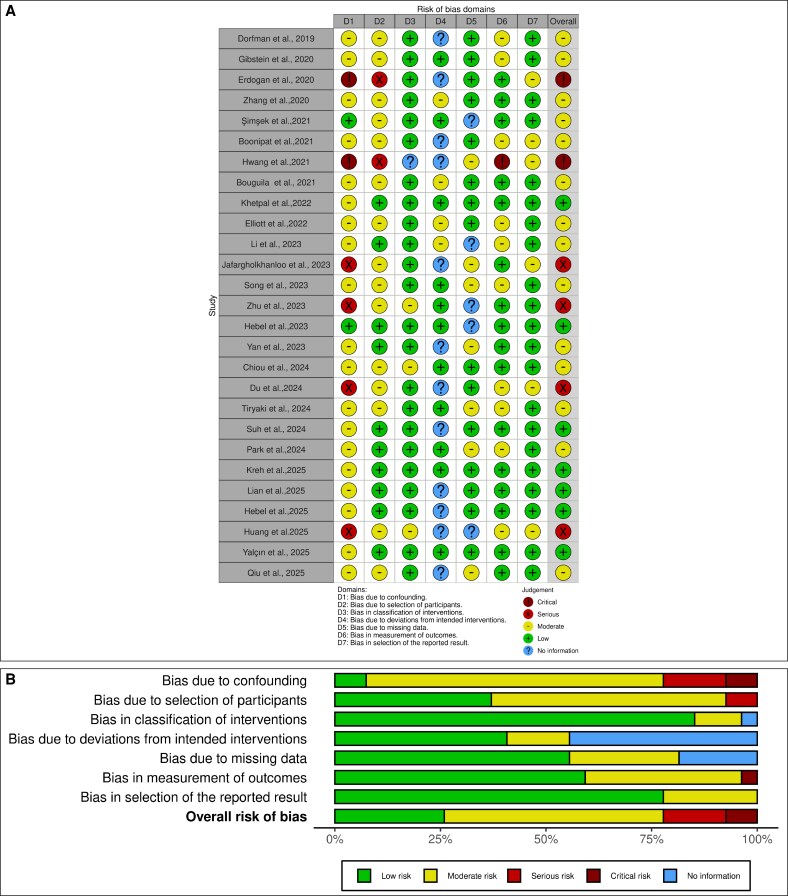
ROBINS-I qualitative assessment of the studies. (A) Traffic-light plot. (B) Summary plot.

**Figure 6:**
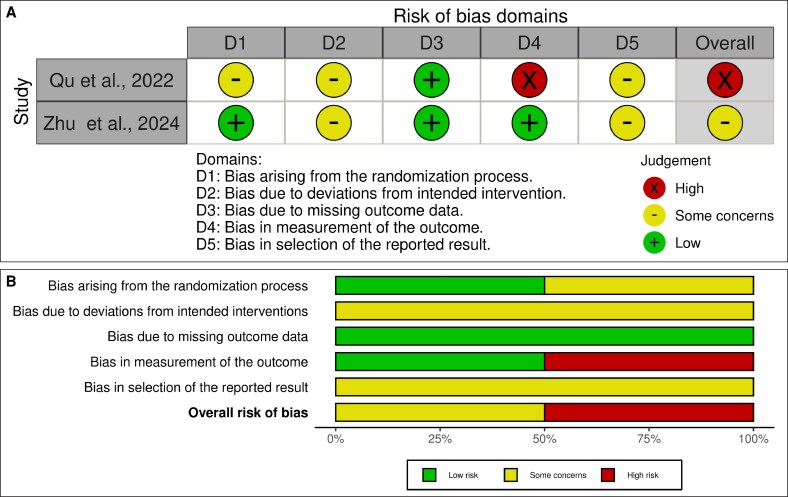
RoB 2 qualitative assessment of the studies. (A) Traffic-light plot. (B) Summary plot.

The overall risk of bias in the nonrandomized studies varied from low risk for 7 studies (25.9%) to moderate risk for 14 studies (51.8%). Two studies (7.4%) were rated critical, and 4 (14.8%) were at serious risk. Major bias reasons included confounding factors, participant selection, missing data, outcome measurement, and reporting biases. The risk of bias in randomized study designs was either moderate or high risk. The detailed bias assessment is presented in Figures 5 and 6.

## DISCUSSION

This systematic review highlights the impact of AI on facial aesthetic surgery outcomes, focusing on applications, clinical stages, and types of procedures. Our review documents a rapid proliferation of AI applications within cosmetic surgery of the cephalic region. However, it indicates that the majority of these applications are derived from feasibility studies. Notably, the most extensively utilized applications belong to outcome assessment and treatment planning. In the former, AI is predominantly employed for age estimation, whereas in treatment planning, it is primarily used for simulation or design processes.

Our findings are consistent with current research on the subject; as with other studies, we found that AI is primarily employed in facial aesthetic surgery for treatment planning and outcome assessment, whereas operational AI remains in its infancy.^5,6^ One reason stems from the data-intensive nature of AI and surgical integration challenges. Compared with preclinical or clinical medical specialties, AI applications in surgical fields (including facial aesthetic surgery) differ from diagnostic applications used in specialties such as pathology, radiology, or dermatology.^59-61^ Further aligning with current findings, CNN-based architectures continue to predominate as the preferred options for AI models, largely because of their capabilities in processing visual data, moderate requirements for datasets, and a more user-friendly ecosystem.^5,6,62^ Our analysis revealed that the majority of AI applications underpin prominent research domains such as facelift, rhinoplasty, and blepharoplasty. These findings align with the latest ISAPS 2024 Global Survey and the 2024 American Society of Plastic Surgeons statistics, with a primary influencing factor being the widespread popularity of these procedures.^4,63^ Of note, only a few included studies actually compared AI with standard care, yet these showed that the evidence is mixed: AI is sometimes noninferior or superior, but also underperforms or contributes meaningfully to assessments.

In a field of rapid change and continuous growth in the research literature, this systematic review offers the latest update on AI's impact on facial aesthetic surgery and addresses gaps in multidisciplinary and transdisciplinary expertise. Furthermore, protocol registration in PROSPERO, adherence to PRISMA, risk-of-bias assessment using the well-validated ROBINS-I and RoB 2 tools, along with a strict focus on institutionally sourced clinical data, ensure the robustness of this systematic review.

As with all systematic reviews, this study has several limitations. Firstly, it was not feasible to conduct a meta-analysis because of the heterogeneity of study designs and various outcome assessment methods. Secondly, excluding AI-specific databases, gray literature, clinical trials, or non-English articles may have limited the ability to capture relevant studies.

Thirdly, studies related to large language models (LLMs) did not meet the inclusion criteria, because we focused on AI applications derived from clinical data (eg, patient images and 3D data). Although LLMs are increasingly used in medicine, their primary applications for cosmetic surgery have been as conversational agents for patient education or simulated consultations or for generating patient communications, rather than as validated perioperative decision-support systems linked to surgical data and outcomes.^64-67^ Future research would also be needed to evaluate how these LLM-based contributions could potentially enhance surgical outcomes and planning.

Some limitations originate from the primary studies themselves. One key limitation is the geographic concentration of the evidence, mainly from Asia and the United States, with sparse data on patients from Europe, Latin America, or sub-Saharan Africa. This may limit the review's cross-cultural generalizability, as facial features, beauty standards, and surgical goals differ across populations.^68^ It was also not possible to perform subgroup analysis by region or ethnicity because of a lack of comparability across heterogeneous AI tasks and cosmetic surgery outcome metrics. Additionally, a persistent challenge in the field is that AI applications inherit potential human demographic and racial biases from training data, which may lead to systematic bias in surgical planning and applications, such as in age estimation algorithms.^69,70^

Given the majority of included studies were feasibility studies, we were unable to assess the extent to which AI could add value over and above the performance of medical professionals in real-world settings. Our findings may be affected by publication bias and exclusion criteria, particularly in surgical AI research, where positive results are more likely to be published.^71^ As a result, the studies included may overrepresent favorable AI performance and clinical impact. A quantitative assessment, such as funnel plot analysis, was unsuitable because of heterogeneity and the absence of a common effect estimate.

### Implications for Future Research

Notably, the research included in this review was conducted in Asian countries (*n* = 13), followed by the United States (*n* = 11), across multicenter or single-center studies. Research should also be conducted in other leading cosmetic surgery countries that perform millions of procedures annually, such as Brazil, India, and European countries, as shown by the ISAPS Global Survey 2024.^4^ Current study designs include mostly applicability, technical development, or technical validation studies; future work should focus on clinically randomized, ideally blinded frameworks that employ AI models that are externally validated, transparent, and explainable.

Future research directions should move beyond the feasibility stage and include study designs that allow researchers to control for confounding domains, alongside appropriate inferential statistical modeling. Deployed systems should include auditing, performance reassessment, monitoring for dataset drift, and AI discrepancy reporting. Nonetheless, the cosmetic surgery research community should aim to conduct and report studies based on AI-specifically designed guidelines, such as STARD-AI for diagnostic accuracy, TRIPOD-AI for predictive modeling, CLAIM-AI for medical imaging, or CONSORT-AI and SPIRIT-AI in clinical trials.^72-75^ The integration of AI into the clinical stages of care shall proceed with a balance between the adoption of innovation and risk management.

### Implications for Future Practice and Policy

The findings of this review indicate that AI could hold a promising impact on facial aesthetic surgery, particularly in treatment planning and outcome assessment stages. Medical practitioners should take advantage of the educational programs that familiarize them with AI principles, benefits, and possible sources of bias.^76,77^ This would enable them to work collaboratively with developers to customize AI models that are solution oriented and actively shaped by their end users.

From a medicolegal and ethical standpoint, synthetic AI outputs should be approached to foster a healthy doctor–patient relationship. Regarding AI-powered simulations, outputs should be regarded as illustrative possibilities, not patient-specific predictions, because these could worsen appearance-related distress in vulnerable individuals like those with body dysmorphic disorders. Given the risk of dataset-driven bias, plastic surgeons should critically evaluate the applicability of AI models to patient populations that may not be represented in the original training data. Expectation management challenges also occur with technologies such as 3D systems. Future research should compare AI simulations and papers to foundational work on Crisalix SA (Lausanne, Switzerland) and VECTRA (Canfield Scientific, Inc., NJ), considering factors like cost, customization, learning curve, calibration, ease, accuracy, and efficiency when comparing modalities.^78,79^ Nonetheless, the new AI-powered 3D systems have applications for patient assessment in aesthetic hair and skin procedures, as well as in cosmetic facial surgery.^80-83^

Informed consent should clearly state when AI simulations or predictions are used, explain the probabilistic nature of results, and outline responsibility. Recent articles address AI concerns in healthcare and propose frameworks for redesigning informed consent.^84,85^ Strategies suitable for cosmetic surgery include using explainable AI, handling unrepresentative datasets, managing patient risk awareness, and keeping human oversight over AI outputs. Future research should focus on longitudinal studies to assess the impact of redesigned consents on patient understanding and healthcare outcomes.

## CONCLUSIONS

Our systematic review of AI's impact on facial aesthetic surgery underscores its potential, particularly in outcome assessment and treatment planning, with facelift, rhinoplasty, and blepharoplasty being the most studied procedures. Most included studies were feasibility studies, whereas a few comparative studies against standard care showed mixed results. Overall, the evidence indicates that AI is an emerging tool in this field, although current clinical data are limited and would benefit from more robust comparative and externally validated research. Given the complexity of aesthetic surgery, continued research into AI technologies may improve the quality of care.

## Supplemental Material

This article contains supplemental material located online at https://doi.org/10.1093/asjof/ojag114.

## Supplementary Material

ojag114_Supplementary_Data
