# Supplementary material for: The Impact of Artificial Intelligence on Facial Aesthetic Surgery: A Systematic Review
Source: Aesthet Surg J Open Forum. 2026 Jun 23;8:ojag114. doi: 10.1093/asjof/ojag114 (PMC13345743; doi:10.1093/asjof/ojag114)
Supplement: ojag114_Supplementary_Data [file ojag114_supplementary_data.zip › Appendix 1.docx]

| **PUBMED Research Query**  **n= 622** | | | | |
| --- | --- | --- | --- | --- |
| **ARTIFICIAL INTELLIGENCE** | **BODY**  **COSMETIC SURGERY** | | **HEAD +NECK**  **COSMETICSURGERY** | |
| **#1** | **#2** | **#3** | **#4** | **#5** |
|  | **#1 AND #2 = #6** | **#1 AND #3 = #7** | **#1 AND #4= #8** | **#1 AND #5= #9** |
| "AI"[Title/Abstract] OR "artificial* intelligen*"[Title/Abstract] OR "machine intelligence"[Title/Abstract] OR "comput* intelligen*" OR"cognitive computing"[Title/Abstract] OR "XAI"[Title/Abstract] OR "Machine Learning"[Title/Abstract] OR "Deep Learning"[Title/Abstract] OR "Language model*"[Title/Abstract] OR "LLM"[Title/Abstract] OR "Supervised learning"[Title/Abstract] OR "Reinforcement learning"[Title/Abstract] OR "reactive machine*"[Title/Abstract] OR "KNN"[Title/Abstract] OR "k nearest neighbour*"[Title/Abstract] OR "k-NN*"[Title/Abstract] OR "k-nearest neighbour*"OR "k-means"[Title/Abstract] OR "support vector* machine*"[Title/Abstract] OR "SVM"[Title/Abstract] OR "random forest*"[Title/Abstract] OR "transfer learning"[Title/Abstract] OR "naive bayes"[Title/Abstract] OR "LSTM"[Title/Abstract] OR "natural language process*"[Title/Abstract] OR "natural language generat*"OR "natural language understand*"[Title/Abstract] OR "relationship extraction"[Title/Abstract] OR "named entity normalization"[Title/Abstract] OR "named entity recognition"[Title/Abstract] OR "text segmentation"[Title/Abstract] OR "tokenization*"[Title/Abstract] OR "lemmatization*"[Title/Abstract] OR "topic* modelling"[Title/Abstract] OR "keyword extraction*"[Title/Abstract] OR "text classification*"OR "text summarization*"OR "sentiment analysis"OR "topic modeling*"OR "machine translation*"OR "word embedd*"OR "stop* word* removal*"[Title/Abstract] OR "neural network*"[Title/Abstract] OR "CNN"[Title/Abstract] OR "DNN"[Title/Abstract] OR "RNN"[Title/Abstract] OR "FNN"[Title/Abstract] OR "ANN"[Title/Abstract] OR "deep belief network*"[Title/Abstract] OR "generative adversial network*"[Title/Abstract] OR "GANs"[Title/Abstract] OR "transform* network*"[Title/Abstract] OR "radial basis function network*"[Title/Abstract] OR "autoencoder*"[Title/Abstract] OR "gated reccurent unit*"[Title/Abstract] OR "autonomous* robot*"[Title/Abstract] OR "artificial* intelligen* robot*"[Title/Abstract] OR "AI robot*"[Title/Abstract] OR "explainable robot*"[Title/Abstract] OR "fuzzy logic*"OR "fuzzy method*"[Title/Abstract] OR "machine vision"[Title/Abstract] OR "comput* vision*"[Title/Abstract] OR "comput* reason*"[Title/Abstract] OR "expert system*"[Title/Abstract] OR "recommender system*"[Title/Abstract] OR "intelligent system*"[Title/Abstract] OR "intelligent agent*"[Title/Abstract] OR "intelligent support"[Title/Abstract] OR "virtual* assist*"[Title/Abstract] OR "chatbot*"[Title/Abstract] OR "chat bot*"[Title/Abstract] OR "GPT"[Title/Abstract] OR "emotion* analysis"[Title/Abstract] OR "opinion mining"[Title/Abstract] OR "data mining"[Title/Abstract] OR "intelligen* analyt*"[Title/Abstract] OR "intelligen* predict*"[Title/Abstract] OR "intelligen* recognit*"[Title/Abstract] OR "intelligen* analys*"[Title/Abstract] OR "digital assist*"[Title/Abstract] OR "intelligen* signal process*"[Title/Abstract] OR "big data process*"[Title/Abstract] OR "information process*"[Title/Abstract] OR "speech recognit*"OR "biometric identif*"[Title/Abstract] OR "facial recognit*"[Title/Abstract] OR "face recognit*"[Title/Abstract] OR "computer security"[Title/Abstract] OR "authentication*"[Title/Abstract] OR "pattern recognit*"[Title/Abstract] OR "auto* pattern* recognit*"[Title/Abstract] OR " pattern* recognit* auto*"[Title/Abstract] OR "predict* model*"[Title/Abstract] OR "intelligen* syst*"[Title/Abstract] OR "intelligen* tool*"[Title/Abstract] OR "intelligen* model*"[Title/Abstract] OR "intelligen* comput*"[Title/Abstract] OR "intelligen* detect*"[Title/Abstract] OR "intelligen* interpret*"[Title/Abstract] OR "intelligen* risk*"[Title/Abstract] OR "intelligen* screen*"[Title/Abstract] OR "intelligen* assess*"[Title/Abstract] OR "imag* process*"[Title/Abstract] OR "intelligen* simulat*"[Title/Abstract] OR "intelligen* AR"[Title/Abstract] OR "intelligen* VR"[Title/Abstract] OR "intelligen* virtual* "[Title/Abstract] OR " intelligen* augment* realit*"[Title/Abstract] OR "intelligen* digital"[Title/Abstract] OR "Java"[Title/Abstract] OR "Phyton"[Title/Abstract] OR "Julia"[Title/Abstract] OR "R programm*"[Title/Abstract] OR "R cod*"[Title/Abstract] OR "R language*"[Title/Abstract] OR "C language*"[Title/Abstract] OR "C programm*"[Title/Abstract] OR "C cod*"[Title/Abstract] OR "C language*"[Title/Abstract] | "cosmetic breast implant*"[Title/Abstract] OR "aesthetic breast implant*"[Title/Abstract] OR "elective breast implant*"[Title/Abstract] OR "cosmetic breast surg*"[Title/Abstract] OR "aesthetic breast surg*"[Title/Abstract] OR "elective breast surg*"[Title/Abstract] OR "elective breast aug*"[Title/Abstract] OR "cosmetic breast aug*"[Title/Abstract] OR "aesthetic breast aug*"[Title/Abstract] OR "breast lift*"[Title/Abstract] OR "breast* symmetrization*"[Title/Abstract] OR "cosmetic mastopex*"[Title/Abstract] OR "aesthetic mastopex*"[Title/Abstract] OR "elective mastopex*"[Title/Abstract] OR "aesthetic breast reduct*"[Title/Abstract] OR "cosmetic breast reduct*"[Title/Abstract] OR "elective breast reduct*"[Title/Abstract] OR "mammaplast*"[Title/Abstract] OR "mammoplast*"[Title/Abstract] OR "abdominoplast*"[Title/Abstract] OR "cosmetic abdominoplast*"[Title/Abstract] OR "aesthetic abdominoplast*"[Title/Abstract] OR "elective abdominoplast*"[Title/Abstract] OR "tumm* tuck*"[Title/Abstract] OR "panniculectom*"[Title/Abstract] OR "lipoabdominoplast*"[Title/Abstract] OR "momm* makeover*"[Title/Abstract] OR "body contour*"[Title/Abstract] OR "fat suction*"[Title/Abstract] OR "abdominal etching"[Title/Abstract] OR "liposuction*"[Title/Abstract] OR "cosmetic liposuct*"[Title/Abstract] OR "aesthetic liposuct*"[Title/Abstract] OR "elective liposuct*"[Title/Abstract] OR "aesthetic contour*"[Title/Abstract] OR "body contour* surg*"[Title/Abstract] OR "surg* body contour*"[Title/Abstract] OR "lipectom*"[Title/Abstract] OR "liposculptur*"[Title/Abstract] OR "lipoplast*"[Title/Abstract] OR "lift* surg*"[Title/Abstract] OR "mons susp*"[Title/Abstract] OR "mons pubis susp*"[Title/Abstract] OR "mons lipo*"[Title/Abstract] OR "mons pubis lipo*"[Title/Abstract] OR "mons pubis reduct*"[Title/Abstract] OR "cosmetic monsplast*"[Title/Abstract] OR "pubic lift*"[Title/Abstract] OR "pubic contour*"[Title/Abstract] OR "limb contour*"[Title/Abstract] OR "extremit* contour*"[Title/Abstract] OR "gluteal augmentation*"OR "gluteal implant*"[Title/Abstract] OR "gluteoplast*"[Title/Abstract] OR "Brazilian butt lift"[Title/Abstract] OR "gluteal reduct*"[Title/Abstract] OR "gluteal lift*"[Title/Abstract] OR "buttock* implant*"[Title/Abstract] OR "brachioplast*"[Title/Abstract] OR "arm contour*"[Title/Abstract] OR "upper limb* contour*"[Title/Abstract] OR "cosmetic arm*"[Title/Abstract] OR "aesthetic arm*"[Title/Abstract] OR "upper arm lift*"[Title/Abstract] OR "biceps* implant*"[Title/Abstract] OR "deltoid implant*"[Title/Abstract] | "thighplast*"[Title/Abstract] OR "aesthetic lower limb*"[Title/Abstract] OR "cosmetic lower limb*"[Title/Abstract] OR "thigh lift*"[Title/Abstract] OR "thigh lipo*"[Title/Abstract] OR "aesthetic calf"[Title/Abstract] OR "cosmetic calf"[Title/Abstract] OR "calf implant*"[Title/Abstract] OR "calf reduct*"[Title/Abstract] OR "calf aug*"[Title/Abstract] OR "leg contour*"[Title/Abstract] OR "extremit* contour*"[Title/Abstract] OR "lower limb* contour*"[Title/Abstract] OR "aesthetic leg*"[Title/Abstract] OR "cosmetic leg*"[Title/Abstract] OR "cosmetic implant*"[Title/Abstract] OR "aesthetic implant*"[Title/Abstract] OR "aesthetic body implant*"[Title/Abstract] OR "aesthetic genit* surg*"[Title/Abstract] OR "cosmetic genit* surg*"[Title/Abstract] OR "genital cosmetic surg*"[Title/Abstract] OR "genital aesthetic surg*"[Title/Abstract] OR "genit* rejuv*"[Title/Abstract] OR "cosmetic gynecology"[Title/Abstract] OR "vulvovaginal aesthetic surg*"[Title/Abstract] OR "aesthetic labiaplast*"[Title/Abstract] OR "cosmetic labiaplast*"[Title/Abstract] OR "cosmetic labioplast*"[Title/Abstract] OR "aesthetic labioplast*"[Title/Abstract] OR "aesthetic labia minora reduct*"[Title/Abstract] OR "cosmetic labia minora reduct*"[Title/Abstract] OR "aesthetic labial reduct*"[Title/Abstract] OR "cosmetic labial reduct*"[Title/Abstract] OR "labia majora reduct*"[Title/Abstract] OR "majoraplast*"[Title/Abstract] OR "labia majora aug*"[Title/Abstract] OR "gynecomastia surg*"[Title/Abstract] OR "pectoral implant"[Title/Abstract] OR "scrot* rejuv*"[Title/Abstract] OR "cosmetic urolog*"[Title/Abstract] OR "aesthetic scrotoplast*"[Title/Abstract] OR "cosmetic phalloplast*"[Title/Abstract] OR "aesthetic phalloplast*"[Title/Abstract] OR "cosmetic scrotoplast*"[Title/Abstract] OR "aesthetic scrotoplast*"[Title/Abstract] OR "cosmetic penoplast*"[Title/Abstract] OR "aesthetic penoplast*"[Title/Abstract] OR "scrot* lift*"[Title/Abstract] OR "cosmetic* surg*"[Title/Abstract] OR "aesthetic* surg*"[Title/Abstract] OR "esthetic* surg*"[Title/Abstract] OR "cosmetic* plastic surg*"[Title/Abstract] OR "aesthetic* plastic surg*"[Title/Abstract] OR "esthetic* plastic surg*"[Title/Abstract] OR "elective plastic surg*"[Title/Abstract] OR "breast* implant*"[Title/Abstract] OR "breast* reduct*"[Title/Abstract] OR "breast* aug*"[Title/Abstract] OR "mastopex*"[Title/Abstract] | "hair transplant*"[Title/Abstract] OR "follicular unit transplant*"[Title/Abstract] OR "surgical hair restoration*"[Title/Abstract] OR "hair* implant*"[Title/Abstract] OR "cosmetic rhinoplast*"[Title/Abstract] OR "aesthetic rhinoplast*"[Title/Abstract] OR "elective rhinoplast*"[Title/Abstract] OR "cosmetic septorhinoplast*"[Title/Abstract] OR "aesthetic septorhinoplast*"[Title/Abstract] OR "elective septorhinoplast*"[Title/Abstract] OR "tip plasty"[Title/Abstract] OR "aesthetic rhinoseptoplast*"[Title/Abstract] OR "cosmetic rhinoseptoplast*"[Title/Abstract] OR "elective rhinoseptoplast*"[Title/Abstract] OR "ethnic rhinoplast*"[Title/Abstract] OR "nasal reduct*"[Title/Abstract] OR "nose reduct*"[Title/Abstract] OR "nasal aug*"[Title/Abstract] OR "nose aug*"[Title/Abstract] OR "nasal *shap*"[Title/Abstract] OR "aesthetic nose surg*"[Title/Abstract] OR "cosmetic nasal surg*"[Title/Abstract] OR "aesthetic nasal surg*"[Title/Abstract] OR "blepharoplast*"[Title/Abstract] OR "cosmetic* blepharoplast*"[Title/Abstract] OR "aesthetic* blepharoplast*"[Title/Abstract] OR "elective blepharoplast*"[Title/Abstract] OR "cosmetic eye* surg*"[Title/Abstract] OR "aesthetic eye* surg*"[Title/Abstract] OR "elective eye* surg*"[Title/Abstract] OR "cosmetic oculoplast*"[Title/Abstract] OR "aesthetic oculoplast*"[Title/Abstract] OR "elective oculoplast*"[Title/Abstract] OR "double eyelid* surg*"[Title/Abstract] OR "canthopex*"[Title/Abstract] OR "canthal susp*"[Title/Abstract] OR "epicanthoplast*"[Title/Abstract] OR "eye rejuv*"[Title/Abstract] OR "browplast*"[Title/Abstract] OR "brow lift*"[Title/Abstract] OR "brow susp*"[Title/Abstract] OR "brow surg*"[Title/Abstract] OR "browpex*"[Title/Abstract] OR "rhytidectom*"[Title/Abstract] OR "rhytidoplast*"[Title/Abstract] OR "facial lift*"[Title/Abstract] OR "face lift*"[Title/Abstract] OR "facelift*"[Title/Abstract] OR "facial rejuv*"[Title/Abstract] OR "forehead lift*"[Title/Abstract] | "temporoplast*"[Title/Abstract] OR "temporal lift*"[Title/Abstract] OR "cheek lift*"[Title/Abstract] OR "malar lift*"[Title/Abstract] OR "neck lift*"[Title/Abstract] OR "neck rejuv*"[Title/Abstract] OR "plastysmaplast*"[Title/Abstract] OR "cervicoplast*"[Title/Abstract] OR "surg* fac* rejuv*"[Title/Abstract] OR "surg* neck* rejuv*"[Title/Abstract] OR "cosmetic *fac* surg*"[Title/Abstract] OR "aesthetic *fac* surg*"[Title/Abstract] OR "elective fac* surg*"[Title/Abstract] OR "aesthetic neck surg*"[Title/Abstract] OR "lip lift*"[Title/Abstract] OR "corner lift*"[Title/Abstract] OR "lip surg*"[Title/Abstract] OR "surg* lip* aug*"[Title/Abstract] OR "augm* lip"[Title/Abstract] OR "lip reduct*"[Title/Abstract] OR "aesthetic ear* surg*"[Title/Abstract] OR "cosmetic ear* surg*"[Title/Abstract] OR "aesthetic otoplast*"[Title/Abstract] OR "cosmetic otoplast*"[Title/Abstract] OR "ear rejuv*"[Title/Abstract] OR "pinnaplast*"[Title/Abstract] OR "ear pinning"[Title/Abstract] OR "ear reduct*"[Title/Abstract] OR "mentoplast*"[Title/Abstract] OR "aesthetic chin surg*"[Title/Abstract] OR "cosmetic chin surg*"[Title/Abstract] OR "aesthetic genioplast*"[Title/Abstract] OR "cosmetic genioplast*"[Title/Abstract] OR "chin *shap*"[Title/Abstract] OR "chin augment*"[Title/Abstract] OR "chin reduct*"[Title/Abstract] OR "jaw *shap*"[Title/Abstract] OR "jaw augment*"[Title/Abstract] OR "jaw reduct*"[Title/Abstract] OR "face reshap*"[Title/Abstract] OR "facial reshap*"[Title/Abstract] OR "facial *shap* surg*"[Title/Abstract] OR "face *shap* surg*"[Title/Abstract] OR "facial implant*"[Title/Abstract] OR "bichectom*"[Title/Abstract] OR "profiloplast*"[Title/Abstract] OR "profileplast*"[Title/Abstract] OR "cheek *shap*"[Title/Abstract] OR "malar *shap*"[Title/Abstract] OR "cheek aug*"[Title/Abstract] OR "malar aug*"[Title/Abstract] OR "malarplast*"[Title/Abstract] OR "malar plast*"[Title/Abstract] OR "facial *suction*"[Title/Abstract] OR "face *suction*"[Title/Abstract] OR "neck liposuct*"[Title/Abstract] OR "neck contour*"[Title/Abstract] OR "facial implant*"[Title/Abstract] OR "face implant*"[Title/Abstract] OR "cosmetic facial implant*"[Title/Abstract] OR "aesthetic facial implant*"[Title/Abstract] OR "temporal implant*"[Title/Abstract] OR "frontal implant*"[Title/Abstract] OR "lip implant*"[Title/Abstract] OR "nose implant*"[Title/Abstract] OR "nasal implant*"[Title/Abstract] OR "jawimplant*"[Title/Abstract] OR "chin implant*"[Title/Abstract] OR "mandib* angle* implant*"[Title/Abstract] OR "malar implant*"[Title/Abstract] OR "cheek* implant*"[Title/Abstract] OR "zygoma* implant*"[Title/Abstract] OR "rhinoplast*"OR "otoplast*"[Title/Abstract] OR "facial asymmetr*"[Title/Abstract] OR "face asymmetr*"[Title/Abstract] OR "elective *fac* surg*"[Title/Abstract] OR "jaw surg*"[Title/Abstract] |

| **#6** | **n=128** | ("AI"[Title/Abstract] OR "artificial* intelligen*"[Title/Abstract] OR "machine intelligence"[Title/Abstract] OR "comput* intelligen*" OR"cognitive computing"[Title/Abstract] OR "XAI"[Title/Abstract] OR "Machine Learning"[Title/Abstract] OR "Deep Learning"[Title/Abstract] OR "Language model*"[Title/Abstract] OR "LLM"[Title/Abstract] OR "Supervised learning"[Title/Abstract] OR "Reinforcement learning"[Title/Abstract] OR "reactive machine*"[Title/Abstract] OR "KNN"[Title/Abstract] OR "k nearest neighbour*"[Title/Abstract] OR "k-NN*"[Title/Abstract] OR "k-nearest neighbour*"OR "k-means"[Title/Abstract] OR "support vector* machine*"[Title/Abstract] OR "SVM"[Title/Abstract] OR "random forest*"[Title/Abstract] OR "transfer learning"[Title/Abstract] OR "naive bayes"[Title/Abstract] OR "LSTM"[Title/Abstract] OR "natural language process*"[Title/Abstract] OR "natural language generat*"OR "natural language understand*"[Title/Abstract] OR "relationship extraction"[Title/Abstract] OR "named entity normalization"[Title/Abstract] OR "named entity recognition"[Title/Abstract] OR "text segmentation"[Title/Abstract] OR "tokenization*"[Title/Abstract] OR "lemmatization*"[Title/Abstract] OR "topic* modelling"[Title/Abstract] OR "keyword extraction*"[Title/Abstract] OR "text classification*"OR "text summarization*"OR "sentiment analysis"OR "topic modeling*"OR "machine translation*"OR "word embedd*"OR "stop* word* removal*"[Title/Abstract] OR "neural network*"[Title/Abstract] OR "CNN"[Title/Abstract] OR "DNN"[Title/Abstract] OR "RNN"[Title/Abstract] OR "FNN"[Title/Abstract] OR "ANN"[Title/Abstract] OR "deep belief network*"[Title/Abstract] OR "generative adversial network*"[Title/Abstract] OR "GANs"[Title/Abstract] OR "transform* network*"[Title/Abstract] OR "radial basis function network*"[Title/Abstract] OR "autoencoder*"[Title/Abstract] OR "gated reccurent unit*"[Title/Abstract] OR "autonomous* robot*"[Title/Abstract] OR "artificial* intelligen* robot*"[Title/Abstract] OR "AI robot*"[Title/Abstract] OR "explainable robot*"[Title/Abstract] OR "fuzzy logic*"OR "fuzzy method*"[Title/Abstract] OR "machine vision"[Title/Abstract] OR "comput* vision*"[Title/Abstract] OR "comput* reason*"[Title/Abstract] OR "expert system*"[Title/Abstract] OR "recommender system*"[Title/Abstract] OR "intelligent system*"[Title/Abstract] OR "intelligent agent*"[Title/Abstract] OR "intelligent support"[Title/Abstract] OR "virtual* assist*"[Title/Abstract] OR "chatbot*"[Title/Abstract] OR "chat bot*"[Title/Abstract] OR "GPT"[Title/Abstract] OR "emotion* analysis"[Title/Abstract] OR "opinion mining"[Title/Abstract] OR "data mining"[Title/Abstract] OR "intelligen* analyt*"[Title/Abstract] OR "intelligen* predict*"[Title/Abstract] OR "intelligen* recognit*"[Title/Abstract] OR "intelligen* analys*"[Title/Abstract] OR "digital assist*"[Title/Abstract] OR "intelligen* signal process*"[Title/Abstract] OR "big data process*"[Title/Abstract] OR "information process*"[Title/Abstract] OR "speech recognit*"OR "biometric identif*"[Title/Abstract] OR "facial recognit*"[Title/Abstract] OR "face recognit*"[Title/Abstract] OR "computer security"[Title/Abstract] OR "authentication*"[Title/Abstract] OR "pattern recognit*"[Title/Abstract] OR "auto* pattern* recognit*"[Title/Abstract] OR " pattern* recognit* auto*"[Title/Abstract] OR "predict* model*"[Title/Abstract] OR "intelligen* syst*"[Title/Abstract] OR "intelligen* tool*"[Title/Abstract] OR "intelligen* model*"[Title/Abstract] OR "intelligen* comput*"[Title/Abstract] OR "intelligen* detect*"[Title/Abstract] OR "intelligen* interpret*"[Title/Abstract] OR "intelligen* risk*"[Title/Abstract] OR "intelligen* screen*"[Title/Abstract] OR "intelligen* assess*"[Title/Abstract] OR "imag* process*"[Title/Abstract] OR "intelligen* simulat*"[Title/Abstract] OR "intelligen* AR"[Title/Abstract] OR "intelligen* VR"[Title/Abstract] OR "intelligen* virtual* "[Title/Abstract] OR " intelligen* augment* realit*"[Title/Abstract] OR "intelligen* digital"[Title/Abstract] OR "Java"[Title/Abstract] OR "Phyton"[Title/Abstract] OR "Julia"[Title/Abstract] OR "R programm*"[Title/Abstract] OR "R cod*"[Title/Abstract] OR "R language*"[Title/Abstract] OR "C language*"[Title/Abstract] OR "C programm*"[Title/Abstract] OR "C cod*"[Title/Abstract] OR "C language*"[Title/Abstract]) AND ("cosmetic breast implant*"[Title/Abstract] OR "aesthetic breast implant*"[Title/Abstract] OR "elective breast implant*"[Title/Abstract] OR "cosmetic breast surg*"[Title/Abstract] OR "aesthetic breast surg*"[Title/Abstract] OR "elective breast surg*"[Title/Abstract] OR "elective breast aug*"[Title/Abstract] OR "cosmetic breast aug*"[Title/Abstract] OR "aesthetic breast aug*"[Title/Abstract] OR "breast lift*"[Title/Abstract] OR "breast* symmetrization*"[Title/Abstract] OR "cosmetic mastopex*"[Title/Abstract] OR "aesthetic mastopex*"[Title/Abstract] OR "elective mastopex*"[Title/Abstract] OR "aesthetic breast reduct*"[Title/Abstract] OR "cosmetic breast reduct*"[Title/Abstract] OR "elective breast reduct*"[Title/Abstract] OR "mammaplast*"[Title/Abstract] OR "mammoplast*"[Title/Abstract] OR "abdominoplast*"[Title/Abstract] OR "cosmetic abdominoplast*"[Title/Abstract] OR "aesthetic abdominoplast*"[Title/Abstract] OR "elective abdominoplast*"[Title/Abstract] OR "tumm* tuck*"[Title/Abstract] OR "panniculectom*"[Title/Abstract] OR "lipoabdominoplast*"[Title/Abstract] OR "momm* makeover*"[Title/Abstract] OR "body contour*"[Title/Abstract] OR "fat suction*"[Title/Abstract] OR "abdominal etching"[Title/Abstract] OR "liposuction*"[Title/Abstract] OR "cosmetic liposuct*"[Title/Abstract] OR "aesthetic liposuct*"[Title/Abstract] OR "elective liposuct*"[Title/Abstract] OR "aesthetic contour*"[Title/Abstract] OR "body contour* surg*"[Title/Abstract] OR "surg* body contour*"[Title/Abstract] OR "lipectom*"[Title/Abstract] OR "liposculptur*"[Title/Abstract] OR "lipoplast*"[Title/Abstract] OR "lift* surg*"[Title/Abstract] OR "mons susp*"[Title/Abstract] OR "mons pubis susp*"[Title/Abstract] OR "mons lipo*"[Title/Abstract] OR "mons pubis lipo*"[Title/Abstract] OR "mons pubis reduct*"[Title/Abstract] OR "cosmetic monsplast*"[Title/Abstract] OR "pubic lift*"[Title/Abstract] OR "pubic contour*"[Title/Abstract] OR "limb contour*"[Title/Abstract] OR "extremit* contour*"[Title/Abstract] OR "gluteal augmentation*"OR "gluteal implant*"[Title/Abstract] OR "gluteoplast*"[Title/Abstract] OR "Brazilian butt lift"[Title/Abstract] OR "gluteal reduct*"[Title/Abstract] OR "gluteal lift*"[Title/Abstract] OR "buttock* implant*"[Title/Abstract] OR "brachioplast*"[Title/Abstract] OR "arm contour*"[Title/Abstract] OR "upper limb* contour*"[Title/Abstract] OR "cosmetic arm*"[Title/Abstract] OR "aesthetic arm*"[Title/Abstract] OR "upper arm lift*"[Title/Abstract] OR "biceps* implant*"[Title/Abstract] OR "deltoid implant*"[Title/Abstract]) |
| --- | --- | --- |
|  | | |
| **#7** | **n=202** | ("AI"[Title/Abstract] OR "artificial* intelligen*"[Title/Abstract] OR "machine intelligence"[Title/Abstract] OR "comput* intelligen*" OR"cognitive computing"[Title/Abstract] OR "XAI"[Title/Abstract] OR "Machine Learning"[Title/Abstract] OR "Deep Learning"[Title/Abstract] OR "Language model*"[Title/Abstract] OR "LLM"[Title/Abstract] OR "Supervised learning"[Title/Abstract] OR "Reinforcement learning"[Title/Abstract] OR "reactive machine*"[Title/Abstract] OR "KNN"[Title/Abstract] OR "k nearest neighbour*"[Title/Abstract] OR "k-NN*"[Title/Abstract] OR "k-nearest neighbour*"OR "k-means"[Title/Abstract] OR "support vector* machine*"[Title/Abstract] OR "SVM"[Title/Abstract] OR "random forest*"[Title/Abstract] OR "transfer learning"[Title/Abstract] OR "naive bayes"[Title/Abstract] OR "LSTM"[Title/Abstract] OR "natural language process*"[Title/Abstract] OR "natural language generat*"OR "natural language understand*"[Title/Abstract] OR "relationship extraction"[Title/Abstract] OR "named entity normalization"[Title/Abstract] OR "named entity recognition"[Title/Abstract] OR "text segmentation"[Title/Abstract] OR "tokenization*"[Title/Abstract] OR "lemmatization*"[Title/Abstract] OR "topic* modelling"[Title/Abstract] OR "keyword extraction*"[Title/Abstract] OR "text classification*"OR "text summarization*"OR "sentiment analysis"OR "topic modeling*"OR "machine translation*"OR "word embedd*"OR "stop* word* removal*"[Title/Abstract] OR "neural network*"[Title/Abstract] OR "CNN"[Title/Abstract] OR "DNN"[Title/Abstract] OR "RNN"[Title/Abstract] OR "FNN"[Title/Abstract] OR "ANN"[Title/Abstract] OR "deep belief network*"[Title/Abstract] OR "generative adversial network*"[Title/Abstract] OR "GANs"[Title/Abstract] OR "transform* network*"[Title/Abstract] OR "radial basis function network*"[Title/Abstract] OR "autoencoder*"[Title/Abstract] OR "gated reccurent unit*"[Title/Abstract] OR "autonomous* robot*"[Title/Abstract] OR "artificial* intelligen* robot*"[Title/Abstract] OR "AI robot*"[Title/Abstract] OR "explainable robot*"[Title/Abstract] OR "fuzzy logic*"OR "fuzzy method*"[Title/Abstract] OR "machine vision"[Title/Abstract] OR "comput* vision*"[Title/Abstract] OR "comput* reason*"[Title/Abstract] OR "expert system*"[Title/Abstract] OR "recommender system*"[Title/Abstract] OR "intelligent system*"[Title/Abstract] OR "intelligent agent*"[Title/Abstract] OR "intelligent support"[Title/Abstract] OR "virtual* assist*"[Title/Abstract] OR "chatbot*"[Title/Abstract] OR "chat bot*"[Title/Abstract] OR "GPT"[Title/Abstract] OR "emotion* analysis"[Title/Abstract] OR "opinion mining"[Title/Abstract] OR "data mining"[Title/Abstract] OR "intelligen* analyt*"[Title/Abstract] OR "intelligen* predict*"[Title/Abstract] OR "intelligen* recognit*"[Title/Abstract] OR "intelligen* analys*"[Title/Abstract] OR "digital assist*"[Title/Abstract] OR "intelligen* signal process*"[Title/Abstract] OR "big data process*"[Title/Abstract] OR "information process*"[Title/Abstract] OR "speech recognit*"OR "biometric identif*"[Title/Abstract] OR "facial recognit*"[Title/Abstract] OR "face recognit*"[Title/Abstract] OR "computer security"[Title/Abstract] OR "authentication*"[Title/Abstract] OR "pattern recognit*"[Title/Abstract] OR "auto* pattern* recognit*"[Title/Abstract] OR " pattern* recognit* auto*"[Title/Abstract] OR "predict* model*"[Title/Abstract] OR "intelligen* syst*"[Title/Abstract] OR "intelligen* tool*"[Title/Abstract] OR "intelligen* model*"[Title/Abstract] OR "intelligen* comput*"[Title/Abstract] OR "intelligen* detect*"[Title/Abstract] OR "intelligen* interpret*"[Title/Abstract] OR "intelligen* risk*"[Title/Abstract] OR "intelligen* screen*"[Title/Abstract] OR "intelligen* assess*"[Title/Abstract] OR "imag* process*"[Title/Abstract] OR "intelligen* simulat*"[Title/Abstract] OR "intelligen* AR"[Title/Abstract] OR "intelligen* VR"[Title/Abstract] OR "intelligen* virtual* "[Title/Abstract] OR " intelligen* augment* realit*"[Title/Abstract] OR "intelligen* digital"[Title/Abstract] OR "Java"[Title/Abstract] OR "Phyton"[Title/Abstract] OR "Julia"[Title/Abstract] OR "R programm*"[Title/Abstract] OR "R cod*"[Title/Abstract] OR "R language*"[Title/Abstract] OR "C language*"[Title/Abstract] OR "C programm*"[Title/Abstract] OR "C cod*"[Title/Abstract] OR "C language*"[Title/Abstract]) AND ("thighplast*"[Title/Abstract] OR "aesthetic lower limb*"[Title/Abstract] OR "cosmetic lower limb*"[Title/Abstract] OR "thigh lift*"[Title/Abstract] OR "thigh lipo*"[Title/Abstract] OR "aesthetic calf"[Title/Abstract] OR "cosmetic calf"[Title/Abstract] OR "calf implant*"[Title/Abstract] OR "calf reduct*"[Title/Abstract] OR "calf aug*"[Title/Abstract] OR "leg contour*"[Title/Abstract] OR "extremit* contour*"[Title/Abstract] OR "lower limb* contour*"[Title/Abstract] OR "aesthetic leg*"[Title/Abstract] OR "cosmetic leg*"[Title/Abstract] OR "cosmetic implant*"[Title/Abstract] OR "aesthetic implant*"[Title/Abstract] OR "aesthetic body implant*"[Title/Abstract] OR "aesthetic genit* surg*"[Title/Abstract] OR "cosmetic genit* surg*"[Title/Abstract] OR "genital cosmetic surg*"[Title/Abstract] OR "genital aesthetic surg*"[Title/Abstract] OR "genit* rejuv*"[Title/Abstract] OR "cosmetic gynecology"[Title/Abstract] OR "vulvovaginal aesthetic surg*"[Title/Abstract] OR "aesthetic labiaplast*"[Title/Abstract] OR "cosmetic labiaplast*"[Title/Abstract] OR "cosmetic labioplast*"[Title/Abstract] OR "aesthetic labioplast*"[Title/Abstract] OR "aesthetic labia minora reduct*"[Title/Abstract] OR "cosmetic labia minora reduct*"[Title/Abstract] OR "aesthetic labial reduct*"[Title/Abstract] OR "cosmetic labial reduct*"[Title/Abstract] OR "labia majora reduct*"[Title/Abstract] OR "majoraplast*"[Title/Abstract] OR "labia majora aug*"[Title/Abstract] OR "gynecomastia surg*"[Title/Abstract] OR "pectoral implant"[Title/Abstract] OR "scrot* rejuv*"[Title/Abstract] OR "cosmetic urolog*"[Title/Abstract] OR "aesthetic scrotoplast*"[Title/Abstract] OR "cosmetic phalloplast*"[Title/Abstract] OR "aesthetic phalloplast*"[Title/Abstract] OR "cosmetic scrotoplast*"[Title/Abstract] OR "aesthetic scrotoplast*"[Title/Abstract] OR "cosmetic penoplast*"[Title/Abstract] OR "aesthetic penoplast*"[Title/Abstract] OR "scrot* lift*"[Title/Abstract] OR "cosmetic* surg*"[Title/Abstract] OR "aesthetic* surg*"[Title/Abstract] OR "esthetic* surg*"[Title/Abstract] OR "cosmetic* plastic surg*"[Title/Abstract] OR "aesthetic* plastic surg*"[Title/Abstract] OR "esthetic* plastic surg*"[Title/Abstract] OR "elective plastic surg*"[Title/Abstract] OR "breast* implant*"[Title/Abstract] OR "breast* reduct*"[Title/Abstract] OR "breast* aug*"[Title/Abstract] OR "mastopex*"[Title/Abstract]) |
|  | | |
| **#8** | **n=99** | ("AI"[Title/Abstract] OR "artificial* intelligen*"[Title/Abstract] OR "machine intelligence"[Title/Abstract] OR "comput* intelligen*" OR"cognitive computing"[Title/Abstract] OR "XAI"[Title/Abstract] OR "Machine Learning"[Title/Abstract] OR "Deep Learning"[Title/Abstract] OR "Language model*"[Title/Abstract] OR "LLM"[Title/Abstract] OR "Supervised learning"[Title/Abstract] OR "Reinforcement learning"[Title/Abstract] OR "reactive machine*"[Title/Abstract] OR "KNN"[Title/Abstract] OR "k nearest neighbour*"[Title/Abstract] OR "k-NN*"[Title/Abstract] OR "k-nearest neighbour*"OR "k-means"[Title/Abstract] OR "support vector* machine*"[Title/Abstract] OR "SVM"[Title/Abstract] OR "random forest*"[Title/Abstract] OR "transfer learning"[Title/Abstract] OR "naive bayes"[Title/Abstract] OR "LSTM"[Title/Abstract] OR "natural language process*"[Title/Abstract] OR "natural language generat*"OR "natural language understand*"[Title/Abstract] OR "relationship extraction"[Title/Abstract] OR "named entity normalization"[Title/Abstract] OR "named entity recognition"[Title/Abstract] OR "text segmentation"[Title/Abstract] OR "tokenization*"[Title/Abstract] OR "lemmatization*"[Title/Abstract] OR "topic* modelling"[Title/Abstract] OR "keyword extraction*"[Title/Abstract] OR "text classification*"OR "text summarization*"OR "sentiment analysis"OR "topic modeling*"OR "machine translation*"OR "word embedd*"OR "stop* word* removal*"[Title/Abstract] OR "neural network*"[Title/Abstract] OR "CNN"[Title/Abstract] OR "DNN"[Title/Abstract] OR "RNN"[Title/Abstract] OR "FNN"[Title/Abstract] OR "ANN"[Title/Abstract] OR "deep belief network*"[Title/Abstract] OR "generative adversial network*"[Title/Abstract] OR "GANs"[Title/Abstract] OR "transform* network*"[Title/Abstract] OR "radial basis function network*"[Title/Abstract] OR "autoencoder*"[Title/Abstract] OR "gated reccurent unit*"[Title/Abstract] OR "autonomous* robot*"[Title/Abstract] OR "artificial* intelligen* robot*"[Title/Abstract] OR "AI robot*"[Title/Abstract] OR "explainable robot*"[Title/Abstract] OR "fuzzy logic*"OR "fuzzy method*"[Title/Abstract] OR "machine vision"[Title/Abstract] OR "comput* vision*"[Title/Abstract] OR "comput* reason*"[Title/Abstract] OR "expert system*"[Title/Abstract] OR "recommender system*"[Title/Abstract] OR "intelligent system*"[Title/Abstract] OR "intelligent agent*"[Title/Abstract] OR "intelligent support"[Title/Abstract] OR "virtual* assist*"[Title/Abstract] OR "chatbot*"[Title/Abstract] OR "chat bot*"[Title/Abstract] OR "GPT"[Title/Abstract] OR "emotion* analysis"[Title/Abstract] OR "opinion mining"[Title/Abstract] OR "data mining"[Title/Abstract] OR "intelligen* analyt*"[Title/Abstract] OR "intelligen* predict*"[Title/Abstract] OR "intelligen* recognit*"[Title/Abstract] OR "intelligen* analys*"[Title/Abstract] OR "digital assist*"[Title/Abstract] OR "intelligen* signal process*"[Title/Abstract] OR "big data process*"[Title/Abstract] OR "information process*"[Title/Abstract] OR "speech recognit*"OR "biometric identif*"[Title/Abstract] OR "facial recognit*"[Title/Abstract] OR "face recognit*"[Title/Abstract] OR "computer security"[Title/Abstract] OR "authentication*"[Title/Abstract] OR "pattern recognit*"[Title/Abstract] OR "auto* pattern* recognit*"[Title/Abstract] OR " pattern* recognit* auto*"[Title/Abstract] OR "predict* model*"[Title/Abstract] OR "intelligen* syst*"[Title/Abstract] OR "intelligen* tool*"[Title/Abstract] OR "intelligen* model*"[Title/Abstract] OR "intelligen* comput*"[Title/Abstract] OR "intelligen* detect*"[Title/Abstract] OR "intelligen* interpret*"[Title/Abstract] OR "intelligen* risk*"[Title/Abstract] OR "intelligen* screen*"[Title/Abstract] OR "intelligen* assess*"[Title/Abstract] OR "imag* process*"[Title/Abstract] OR "intelligen* simulat*"[Title/Abstract] OR "intelligen* AR"[Title/Abstract] OR "intelligen* VR"[Title/Abstract] OR "intelligen* virtual* "[Title/Abstract] OR " intelligen* augment* realit*"[Title/Abstract] OR "intelligen* digital"[Title/Abstract] OR "Java"[Title/Abstract] OR "Phyton"[Title/Abstract] OR "Julia"[Title/Abstract] OR "R programm*"[Title/Abstract] OR "R cod*"[Title/Abstract] OR "R language*"[Title/Abstract] OR "C language*"[Title/Abstract] OR "C programm*"[Title/Abstract] OR "C cod*"[Title/Abstract] OR "C language*"[Title/Abstract]) AND ("hair transplant*"[Title/Abstract] OR "follicular unit transplant*"[Title/Abstract] OR "surgical hair restoration*"[Title/Abstract] OR "hair* implant*"[Title/Abstract] OR "cosmetic rhinoplast*"[Title/Abstract] OR "aesthetic rhinoplast*"[Title/Abstract] OR "elective rhinoplast*"[Title/Abstract] OR "cosmetic septorhinoplast*"[Title/Abstract] OR "aesthetic septorhinoplast*"[Title/Abstract] OR "elective septorhinoplast*"[Title/Abstract] OR "tip plasty"[Title/Abstract] OR "aesthetic rhinoseptoplast*"[Title/Abstract] OR "cosmetic rhinoseptoplast*"[Title/Abstract] OR "elective rhinoseptoplast*"[Title/Abstract] OR "ethnic rhinoplast*"[Title/Abstract] OR "nasal reduct*"[Title/Abstract] OR "nose reduct*"[Title/Abstract] OR "nasal aug*"[Title/Abstract] OR "nose aug*"[Title/Abstract] OR "nasal *shap*"[Title/Abstract] OR "aesthetic nose surg*"[Title/Abstract] OR "cosmetic nasal surg*"[Title/Abstract] OR "aesthetic nasal surg*"[Title/Abstract] OR "blepharoplast*"[Title/Abstract] OR "cosmetic* blepharoplast*"[Title/Abstract] OR "aesthetic* blepharoplast*"[Title/Abstract] OR "elective blepharoplast*"[Title/Abstract] OR "cosmetic eye* surg*"[Title/Abstract] OR "aesthetic eye* surg*"[Title/Abstract] OR "elective eye* surg*"[Title/Abstract] OR "cosmetic oculoplast*"[Title/Abstract] OR "aesthetic oculoplast*"[Title/Abstract] OR "elective oculoplast*"[Title/Abstract] OR "double eyelid* surg*"[Title/Abstract] OR "canthopex*"[Title/Abstract] OR "canthal susp*"[Title/Abstract] OR "epicanthoplast*"[Title/Abstract] OR "eye rejuv*"[Title/Abstract] OR "browplast*"[Title/Abstract] OR "brow lift*"[Title/Abstract] OR "brow susp*"[Title/Abstract] OR "brow surg*"[Title/Abstract] OR "browpex*"[Title/Abstract] OR "rhytidectom*"[Title/Abstract] OR "rhytidoplast*"[Title/Abstract] OR "facial lift*"[Title/Abstract] OR "face lift*"[Title/Abstract] OR "facelift*"[Title/Abstract] OR "facial rejuv*"[Title/Abstract] OR "forehead lift*"[Title/Abstract]) |
|  | | |
| **#9** | **n=193** | ("AI"[Title/Abstract] OR "artificial* intelligen*"[Title/Abstract] OR "machine intelligence"[Title/Abstract] OR "comput* intelligen*" OR"cognitive computing"[Title/Abstract] OR "XAI"[Title/Abstract] OR "Machine Learning"[Title/Abstract] OR "Deep Learning"[Title/Abstract] OR "Language model*"[Title/Abstract] OR "LLM"[Title/Abstract] OR "Supervised learning"[Title/Abstract] OR "Reinforcement learning"[Title/Abstract] OR "reactive machine*"[Title/Abstract] OR "KNN"[Title/Abstract] OR "k nearest neighbour*"[Title/Abstract] OR "k-NN*"[Title/Abstract] OR "k-nearest neighbour*"OR "k-means"[Title/Abstract] OR "support vector* machine*"[Title/Abstract] OR "SVM"[Title/Abstract] OR "random forest*"[Title/Abstract] OR "transfer learning"[Title/Abstract] OR "naive bayes"[Title/Abstract] OR "LSTM"[Title/Abstract] OR "natural language process*"[Title/Abstract] OR "natural language generat*"OR "natural language understand*"[Title/Abstract] OR "relationship extraction"[Title/Abstract] OR "named entity normalization"[Title/Abstract] OR "named entity recognition"[Title/Abstract] OR "text segmentation"[Title/Abstract] OR "tokenization*"[Title/Abstract] OR "lemmatization*"[Title/Abstract] OR "topic* modelling"[Title/Abstract] OR "keyword extraction*"[Title/Abstract] OR "text classification*"OR "text summarization*"OR "sentiment analysis"OR "topic modeling*"OR "machine translation*"OR "word embedd*"OR "stop* word* removal*"[Title/Abstract] OR "neural network*"[Title/Abstract] OR "CNN"[Title/Abstract] OR "DNN"[Title/Abstract] OR "RNN"[Title/Abstract] OR "FNN"[Title/Abstract] OR "ANN"[Title/Abstract] OR "deep belief network*"[Title/Abstract] OR "generative adversial network*"[Title/Abstract] OR "GANs"[Title/Abstract] OR "transform* network*"[Title/Abstract] OR "radial basis function network*"[Title/Abstract] OR "autoencoder*"[Title/Abstract] OR "gated reccurent unit*"[Title/Abstract] OR "autonomous* robot*"[Title/Abstract] OR "artificial* intelligen* robot*"[Title/Abstract] OR "AI robot*"[Title/Abstract] OR "explainable robot*"[Title/Abstract] OR "fuzzy logic*"OR "fuzzy method*"[Title/Abstract] OR "machine vision"[Title/Abstract] OR "comput* vision*"[Title/Abstract] OR "comput* reason*"[Title/Abstract] OR "expert system*"[Title/Abstract] OR "recommender system*"[Title/Abstract] OR "intelligent system*"[Title/Abstract] OR "intelligent agent*"[Title/Abstract] OR "intelligent support"[Title/Abstract] OR "virtual* assist*"[Title/Abstract] OR "chatbot*"[Title/Abstract] OR "chat bot*"[Title/Abstract] OR "GPT"[Title/Abstract] OR "emotion* analysis"[Title/Abstract] OR "opinion mining"[Title/Abstract] OR "data mining"[Title/Abstract] OR "intelligen* analyt*"[Title/Abstract] OR "intelligen* predict*"[Title/Abstract] OR "intelligen* recognit*"[Title/Abstract] OR "intelligen* analys*"[Title/Abstract] OR "digital assist*"[Title/Abstract] OR "intelligen* signal process*"[Title/Abstract] OR "big data process*"[Title/Abstract] OR "information process*"[Title/Abstract] OR "speech recognit*"OR "biometric identif*"[Title/Abstract] OR "facial recognit*"[Title/Abstract] OR "face recognit*"[Title/Abstract] OR "computer security"[Title/Abstract] OR "authentication*"[Title/Abstract] OR "pattern recognit*"[Title/Abstract] OR "auto* pattern* recognit*"[Title/Abstract] OR " pattern* recognit* auto*"[Title/Abstract] OR "predict* model*"[Title/Abstract] OR "intelligen* syst*"[Title/Abstract] OR "intelligen* tool*"[Title/Abstract] OR "intelligen* model*"[Title/Abstract] OR "intelligen* comput*"[Title/Abstract] OR "intelligen* detect*"[Title/Abstract] OR "intelligen* interpret*"[Title/Abstract] OR "intelligen* risk*"[Title/Abstract] OR "intelligen* screen*"[Title/Abstract] OR "intelligen* assess*"[Title/Abstract] OR "imag* process*"[Title/Abstract] OR "intelligen* simulat*"[Title/Abstract] OR "intelligen* AR"[Title/Abstract] OR "intelligen* VR"[Title/Abstract] OR "intelligen* virtual* "[Title/Abstract] OR " intelligen* augment* realit*"[Title/Abstract] OR "intelligen* digital"[Title/Abstract] OR "Java"[Title/Abstract] OR "Phyton"[Title/Abstract] OR "Julia"[Title/Abstract] OR "R programm*"[Title/Abstract] OR "R cod*"[Title/Abstract] OR "R language*"[Title/Abstract] OR "C language*"[Title/Abstract] OR "C programm*"[Title/Abstract] OR "C cod*"[Title/Abstract] OR "C language*"[Title/Abstract]) AND ("temporoplast*"[Title/Abstract] OR "temporal lift*"[Title/Abstract] OR "cheek lift*"[Title/Abstract] OR "malar lift*"[Title/Abstract] OR "neck lift*"[Title/Abstract] OR "neck rejuv*"[Title/Abstract] OR "plastysmaplast*"[Title/Abstract] OR "cervicoplast*"[Title/Abstract] OR "surg* fac* rejuv*"[Title/Abstract] OR "surg* neck* rejuv*"[Title/Abstract] OR "cosmetic *fac* surg*"[Title/Abstract] OR "aesthetic *fac* surg*"[Title/Abstract] OR "elective fac* surg*"[Title/Abstract] OR "aesthetic neck surg*"[Title/Abstract] OR "lip lift*"[Title/Abstract] OR "corner lift*"[Title/Abstract] OR "lip surg*"[Title/Abstract] OR "surg* lip* aug*"[Title/Abstract] OR "augm* lip"[Title/Abstract] OR "lip reduct*"[Title/Abstract] OR "aesthetic ear* surg*"[Title/Abstract] OR "cosmetic ear* surg*"[Title/Abstract] OR "aesthetic otoplast*"[Title/Abstract] OR "cosmetic otoplast*"[Title/Abstract] OR "ear rejuv*"[Title/Abstract] OR "pinnaplast*"[Title/Abstract] OR "ear pinning"[Title/Abstract] OR "ear reduct*"[Title/Abstract] OR "mentoplast*"[Title/Abstract] OR "aesthetic chin surg*"[Title/Abstract] OR "cosmetic chin surg*"[Title/Abstract] OR "aesthetic genioplast*"[Title/Abstract] OR "cosmetic genioplast*"[Title/Abstract] OR "chin *shap*"[Title/Abstract] OR "chin augment*"[Title/Abstract] OR "chin reduct*"[Title/Abstract] OR "jaw *shap*"[Title/Abstract] OR "jaw augment*"[Title/Abstract] OR "jaw reduct*"[Title/Abstract] OR "face reshap*"[Title/Abstract] OR "facial reshap*"[Title/Abstract] OR "facial *shap* surg*"[Title/Abstract] OR "face *shap* surg*"[Title/Abstract] OR "facial implant*"[Title/Abstract] OR "bichectom*"[Title/Abstract] OR "profiloplast*"[Title/Abstract] OR "profileplast*"[Title/Abstract] OR "cheek *shap*"[Title/Abstract] OR "malar *shap*"[Title/Abstract] OR "cheek aug*"[Title/Abstract] OR "malar aug*"[Title/Abstract] OR "malarplast*"[Title/Abstract] OR "malar plast*"[Title/Abstract] OR "facial *suction*"[Title/Abstract] OR "face *suction*"[Title/Abstract] OR "neck liposuct*"[Title/Abstract] OR "neck contour*"[Title/Abstract] OR "facial implant*"[Title/Abstract] OR "face implant*"[Title/Abstract] OR "cosmetic facial implant*"[Title/Abstract] OR "aesthetic facial implant*"[Title/Abstract] OR "temporal implant*"[Title/Abstract] OR "frontal implant*"[Title/Abstract] OR "lip implant*"[Title/Abstract] OR "nose implant*"[Title/Abstract] OR "nasal implant*"[Title/Abstract] OR "jawimplant*"[Title/Abstract] OR "chin implant*"[Title/Abstract] OR "mandib* angle* implant*"[Title/Abstract] OR "malar implant*"[Title/Abstract] OR "cheek* implant*"[Title/Abstract] OR "zygoma* implant*"[Title/Abstract] OR "rhinoplast*"OR "otoplast*"[Title/Abstract] OR "facial asymmetr*"[Title/Abstract] OR "face asymmetr*"[Title/Abstract] OR "elective *fac* surg*"[Title/Abstract] OR "jaw surg*"[Title/Abstract]) |
|  | | |
| **#10** | **(#6 AND #7 AND #8 AND #9)**  **n=622** | |

| **Web of Science Research Query**  **n= 1.210** | | |
| --- | --- | --- |
| **ARTIFICIAL INTELLIGENCE**  **#1** | **BODY COSMETIC SURGERY**  **#2** | **HEAD+NECK COSMETIC SURGERY**  **#3** |
| "AI" OR "artificial* intelligen*"OR "machine intelligence" OR "comput* intelligen*" OR"cognitive computing" OR "XAI" OR "Machine Learning" OR "Deep Learning" OR "Language model*" OR "LLM" OR "Supervised learning" OR "Reinforcement learning" OR "reactive machine*" OR "KNN" OR "k nearest neighbour*" OR "k-NN*" OR "k-nearest neighbour*"OR "k-means" OR "support vector* machine*" OR "SVM" OR "random forest*" OR "transfer learning" OR "naive bayes" OR "LSTM" OR "natural language process*" OR "natural language generat*"OR "natural language understand*" OR "relationship extraction" OR "named entity normalization" OR "named entity recognition" OR "text segmentation" OR "tokenization*" OR "lemmatization*" OR "topic* modelling" OR "keyword extraction*" OR "text classification*"OR "text summarization*"OR "sentiment analysis"OR "topic modeling*"OR "machine translation*"OR "word embedd*"OR "stop* word* removal*" OR "neural network*" OR "CNN" OR "DNN" OR "RNN" OR "FNN" OR "ANN" OR "deep belief network*" OR "generative adversial network*" OR "GANs" OR "transform* network*" OR "radial basis **function network*" OR "autoencoder*" OR "gated reccurent unit*" OR "autonomous* robot*" OR "artificial* intelligen* robot*" OR "AI robot*" OR "explainable robot*" OR "fuzzy** logic*"OR "fuzzy method*" OR "machine vision" OR "comput* vision*" OR "comput* reason*" OR "expert system*" OR "recommender system*" OR "intelligent system*" OR "intelligent agent*" OR "intelligent support" OR "virtual* assist*" OR "chatbot*" OR "chat bot*" OR "*GPT*" OR "emotion* analysis" OR "opinion mining" OR "data mining" OR "intelligen* analyt*" OR "intelligen* predict*" OR "intelligen* recognit*" OR "intelligen* analys*" OR "digital assist*" OR "intelligen* signal process*" OR "big data process*" OR "information process*" OR "speech recognit*"OR "biometric identif*" OR "facial recognit*" OR "face recognit*" OR "computer security" OR "authentication*" OR "pattern recognit*" OR "auto* pattern* recognit*" OR " pattern* recognit* auto*"  OR "predict* model*" OR "intelligen* syst*" OR "intelligen* tool*" OR "intelligen* model*" OR "intelligen* comput*" OR "intelligen* detect*" OR "intelligen* interpret*" OR "intelligen* risk*" OR "intelligen* screen*" OR "intelligen* assess*" OR "imag* process*" OR "intelligen* simulat*" OR "intelligen* AR" OR "intelligen* VR" OR "intelligen* virtual* " OR " intelligen* augment* realit*" OR "intelligen* digital" OR "Java" OR "Phyton" OR "Julia" OR "R programm*" OR "R cod*" OR "R language*" OR "C language*" OR "C programm*" OR "C cod*" OR "C language*" | "cosmetic breast implant*" OR "aesthetic breast implant*" OR "elective breast implant*" OR "cosmetic breast surg*" OR "aesthetic breast surg*" OR "elective breast surg*" OR "elective breast aug*" OR "cosmetic breast aug*" OR "aesthetic breast aug*" OR "breast lift*" OR "breast* symmetrization*" OR "cosmetic mastopex*" OR "aesthetic mastopex*" OR "elective mastopex*" OR "aesthetic breast reduct*" OR "cosmetic breast reduct*" OR "elective breast reduct*" OR "mammaplast*" OR "mammoplast*" OR "abdominoplast*" OR "cosmetic abdominoplast*" OR "aesthetic abdominoplast*" OR "elective abdominoplast*" OR "tumm* tuck*" OR "panniculectom*" OR "lipoabdominoplast*" OR "momm* makeover*" OR "body contour*" OR "fat suction*" OR "abdominal etching" OR “liposuction*” OR "cosmetic liposuct*" OR "aesthetic liposuct*" OR "elective liposuct*" OR "aesthetic contour*" OR “body contour* surg*” OR “surg* body contour*” OR “lipectom*” OR “liposculptur*” OR "lipoplast*" OR "lift* surg*" OR "mons susp*" OR "mons pubis susp*" OR "mons lipo*" OR "mons pubis lipo*" OR "mons pubis reduct*" OR "cosmetic monsplast*" OR "pubic lift*" OR "pubic contour*" OR "limb contour*" OR "extremit* contour*" OR “gluteal augmentation*”OR "gluteal implant*" OR “gluteoplast*” OR “Brazilian butt lift” OR "gluteal reduct*" OR "gluteal lift*" OR "buttock* implant*" OR “brachioplast*” OR "arm* contour*" OR "upper limb* contour*" OR "cosmetic arm*" OR "aesthetic arm*" OR “upper arm lift*” OR "biceps* implant*" OR "deltoid implant*"  OR “thighplast*" OR "aesthetic lower limb*" OR "cosmetic lower limb*" OR "thigh lift*" OR "thigh lipo*” OR "aesthetic calf" OR "cosmetic calf" OR "calf implant*" OR "calf reduct*" OR "calf aug*" OR "leg contour*" OR "extremit* contour*" OR "lower limb* contour*" OR "aesthetic leg*" OR "cosmetic leg*" OR "cosmetic implant*" OR "aesthetic implant*" OR "aesthetic body implant*" OR "aesthetic genit* surg*" OR "cosmetic genit* surg*" OR "genital cosmetic surg*" OR "genital aesthetic surg*" OR "genit* rejuv*" OR "cosmetic gynecology" OR "vulvovaginal aesthetic surg*" OR "aesthetic labiaplast*" OR "cosmetic labiaplast*" OR "cosmetic labioplast*" OR "aesthetic labioplast*" OR "aesthetic labia minora reduct*" OR "cosmetic labia minora reduct*" OR "aesthetic labial reduct*" OR "cosmetic labial reduct*" OR "labia majora reduct*" OR "majoraplast*" OR "labia majora aug*" OR "gynecomastia surg*" OR "pectoral implant" OR "scrot* rejuv*" OR "cosmetic urolog*" OR "aesthetic scrotoplast*" OR "cosmetic phalloplast*" OR "aesthetic phalloplast*" OR "cosmetic scrotoplast*" OR "aesthetic scrotoplast*" OR "cosmetic penoplast*" OR "aesthetic penoplast*" OR "scrot* lift*" OR "cosmetic* surg*" OR "aesthetic* surg*" OR "esthetic* surg*" OR "cosmetic* plastic surg*" OR "aesthetic* plastic surg*" OR "esthetic* plastic surg*" OR "elective plastic surg*" OR "breast* implant*" OR "breast* reduct*" OR "breast* aug*" OR "mastopex*" | "hair transplant*" OR "follicular unit transplant*" OR "surgical hair restoration*" OR "hair* implant*" OR "cosmetic rhinoplast*" OR "aesthetic rhinoplast*" OR "elective rhinoplast*" OR "cosmetic septorhinoplast*" OR "aesthetic septorhinoplast*" OR "elective septorhinoplast*" OR "tip plasty" OR "aesthetic rhinoseptoplast*" OR "cosmetic rhinoseptoplast*" OR "elective rhinoseptoplast*" OR "ethnic rhinoplast*" OR "nasal reduct*" OR "nose reduct*" OR "nasal aug*" OR "nose aug*" OR "nasal *shap*" OR "aesthetic nose surg*" OR "cosmetic nasal surg*" OR "aesthetic nasal surg*" OR "blepharoplast*" OR "cosmetic* blepharoplast*" OR "aesthetic* blepharoplast*" OR "elective blepharoplast*" OR "cosmetic eye* surg*" OR "aesthetic eye* surg*" OR "elective eye* surg*" OR "cosmetic oculoplast*" OR "aesthetic oculoplast*" OR "elective oculoplast*" OR "double eyelid* surg*" OR "canthopex*" OR "canthal susp*" OR "epicanthoplast*" OR "*eye* rejuv*" OR "browplast*" OR "*brow* lift*" OR "*brow* susp*" OR "*brow* surg*" OR "browpex*" OR "rhytidectom*" OR "rhytidoplast*" OR "facial lift*" OR "face lift*" OR "facelift*" OR "facial rejuv*" OR "forehead lift*" OR "temporoplast*" OR "temporal lift*" OR "cheek lift*" OR "malar lift*" OR "neck lift*" OR "neck rejuv*" OR "plastysmaplast*" OR "cervicoplast*" OR "surg* fac* rejuv*" OR "surg* neck* rejuv*" OR "cosmetic *fac* surg*" OR "aesthetic *fac* surg*" OR "elective fac* surg*" OR "aesthetic neck surg*" OR "lip lift*" OR "corner lift*" OR "lip surg*" OR "surg* lip* aug*" OR "augm* lip" OR "lip reduct*" OR "aesthetic ear* surg*" OR "cosmetic ear* surg*" OR "aesthetic otoplast*" OR "cosmetic otoplast*" OR "ear* rejuv*" OR "pinnaplast*" OR "ear pinning" OR "ear reduct*" OR "mentoplast*" OR "aesthetic chin surg*" OR "cosmetic chin surg*" OR "aesthetic genioplast*" OR "cosmetic genioplast*" OR "chin *shap*" OR "chin augment*" OR "chin reduct*" OR "jaw *shap*" OR "jaw augment*" OR "jaw reduct*" OR "*face reshap*" OR "*facial reshap*" OR "*facial *shap* surg*" OR "*face *shap* surg*" OR "facial implant*" OR "bichectom*" OR "profiloplast*" OR "profileplast*" OR "*cheek *shap*" OR "malar *shap*" OR "cheek aug*" OR "malar aug*" OR "malarplast*" OR "*malar plast*" OR "*facial *suction*" OR "*face *suction*" OR "*neck liposuct*" OR "neck contour*" OR "*facial implant*" OR "*face implant*" OR "cosmetic facial implant*" OR "aesthetic facial implant*" OR "temporal implant*" OR "frontal implant*" OR "lip implant*" OR "nose implant*" OR "nasal implant*" OR "jaw* implant*" OR "chin implant*" OR "mandib* angle* implant*" OR "malar implant*" OR "*cheek* implant*" OR "zygoma* implant*" OR "rhinoplast*"OR "otoplast*" OR "facial asymmetr*" OR  "face asymmetr*" OR "elective *fac* surg*" OR "*jaw surg*" |

| **#1** | **n= 3.020.669** | "AI" OR "artificial* intelligen*" OR "machine intelligence" OR "comput* intelligen*" OR"cognitive computing" OR "XAI" OR "Machine Learning" OR "Deep Learning" OR "Language model*" OR "LLM" OR "Supervised learning" OR "Reinforcement learning" OR "reactive machine*" OR "KNN" OR "k nearest neighbour*" OR "k-NN*" OR "k-nearest neighbour*"OR "k-means" OR "support vector* machine*" OR "SVM" OR "random forest*" OR "transfer learning" OR "naive bayes" OR "LSTM" OR "natural language process*" OR "natural language generat*"OR "natural language understand*" OR "relationship extraction" OR "named entity normalization" OR "named entity recognition" OR "text segmentation" OR "tokenization*" OR "lemmatization*" OR "topic* modelling" OR "keyword extraction*" OR "text classification*"OR "text summarization*"OR "sentiment analysis"OR "topic modeling*"OR "machine translation*"OR "word embedd*"OR "stop* word* removal*" OR "neural network*" OR "CNN" OR "DNN" OR "RNN" OR "FNN" OR "ANN" OR "deep belief network*" OR "generative adversial network*" OR "GANs" OR "transform* network*" OR "radial basis function network*" OR "autoencoder*" OR "gated reccurent unit*" OR "autonomous* robot*" OR "artificial* intelligen* robot*" OR "AI robot*" OR "explainable robot*" OR "fuzzy logic*"OR "fuzzy method*" OR "machine vision" OR "comput* vision*" OR "comput* reason*" OR "expert system*" OR "recommender system*" OR "intelligent system*" OR "intelligent agent*" OR "intelligent support" OR "virtual* assist*" OR "chatbot*" OR "chat bot*" OR "*GPT*" OR "emotion* analysis" OR "opinion mining" OR "data mining" OR "intelligen* analyt*" OR "intelligen* predict*" OR "intelligen* recognit*" OR "intelligen* analys*" OR "digital assist*" OR "intelligen* signal process*" OR "big data process*" OR "information process*" OR "speech recognit*"OR "biometric identif*" OR "facial recognit*" OR "face recognit*" OR "computer security" OR "authentication*" OR "pattern recognit*" OR "auto* pattern* recognit*" OR " pattern* recognit* auto*" OR "predict* model*" OR "intelligen* syst*" OR "intelligen* tool*" OR "intelligen* model*" OR "intelligen* comput*" OR "intelligen* detect*" OR "intelligen* interpret*" OR "intelligen* risk*" OR "intelligen* screen*" OR "intelligen* assess*" OR "imag* process*" OR "intelligen* simulat*" OR "intelligen* AR" OR "intelligen* VR" OR "intelligen* virtual* " OR " intelligen* augment* realit*" OR "intelligen* digital" OR "Java" OR "Phyton" OR "Julia" OR "R programm*" OR "R cod*" OR "R language*" OR "C language*" OR "C programm*" OR "C cod*" OR "C language*" (Topic) |
| --- | --- | --- |
|  | | |
| **#2** | **n= 39.398** | "cosmetic breast implant*" OR "aesthetic breast implant*" OR "elective breast implant*" OR "cosmetic breast surg*" OR "aesthetic breast surg*" OR "elective breast surg*" OR "elective breast aug*" OR "cosmetic breast aug*" OR "aesthetic breast aug*" OR "breast lift*" OR "breast* symmetrization*" OR "cosmetic mastopex*" OR "aesthetic mastopex*" OR "elective mastopex*" OR "aesthetic breast reduct*" OR "cosmetic breast reduct*" OR "elective breast reduct*" OR "mammaplast*" OR "mammoplast*" OR "abdominoplast*" OR "cosmetic abdominoplast*" OR "aesthetic abdominoplast*" OR "elective abdominoplast*" OR "tumm* tuck*" OR "panniculectom*" OR "lipoabdominoplast*" OR "momm* makeover*" OR "body contour*" OR "fat suction*" OR "abdominal etching" OR “liposuction*” OR "cosmetic liposuct*" OR "aesthetic liposuct*" OR "elective liposuct*" OR "aesthetic contour*" OR “body contour* surg*” OR “surg* body contour*” OR “lipectom*” OR “liposculptur*” OR "lipoplast*" OR "lift* surg*" OR "mons susp*" OR "mons pubis susp*" OR "mons lipo*" OR "mons pubis lipo*" OR "mons pubis reduct*" OR "cosmetic monsplast*" OR "pubic lift*" OR "pubic contour*" OR "limb contour*" OR "extremit* contour*" OR “gluteal augmentation*”OR "gluteal implant*" OR “gluteoplast*” OR “Brazilian butt lift” OR "gluteal reduct*" OR "gluteal lift*" OR "buttock* implant*" OR “brachioplast*” OR "arm* contour*" OR "upper limb* contour*" OR "cosmetic arm*" OR "aesthetic arm*" OR “upper arm lift*” OR "biceps* implant*" OR "deltoid implant*" OR “thighplast*" OR "aesthetic lower limb*" OR "cosmetic lower limb*" OR "thigh lift*" OR "thigh lipo*” OR "aesthetic calf" OR "cosmetic calf" OR "calf implant*" OR "calf reduct*" OR "calf aug*" OR "leg contour*" OR "extremit* contour*" OR "lower limb* contour*" OR "aesthetic leg*" OR "cosmetic leg*" OR "cosmetic implant*" OR "aesthetic implant*" OR "aesthetic body implant*" OR "aesthetic genit* surg*" OR "cosmetic genit* surg*" OR "genital cosmetic surg*" OR "genital aesthetic surg*" OR "genit* rejuv*" OR "cosmetic gynecology" OR "vulvovaginal aesthetic surg*" OR "aesthetic labiaplast*" OR "cosmetic labiaplast*" OR "cosmetic labioplast*" OR "aesthetic labioplast*" OR "aesthetic labia minora reduct*" OR "cosmetic labia minora reduct*" OR "aesthetic labial reduct*" OR "cosmetic labial reduct*" OR "labia majora reduct*" OR "majoraplast*" OR "labia majora aug*" OR "gynecomastia surg*" OR "pectoral implant" OR "scrot* rejuv*" OR "cosmetic urolog*" OR "aesthetic scrotoplast*" OR "cosmetic phalloplast*" OR "aesthetic phalloplast*" OR "cosmetic scrotoplast*" OR "aesthetic scrotoplast*" OR "cosmetic penoplast*" OR "aesthetic penoplast*" OR "scrot* lift*" OR "cosmetic* surg*" OR "aesthetic* surg*" OR "esthetic* surg*" OR "cosmetic* plastic surg*" OR "aesthetic* plastic surg*" OR "esthetic* plastic surg*" OR "elective plastic surg*" OR "breast* implant*" OR "breast* reduct*" OR "breast* aug*" OR "mastopex*" (Topic) |
|  | | |
| **#3** | **n= 29.072** | "hair transplant*" OR "follicular unit transplant*" OR "surgical hair restoration*" OR "hair* implant*" OR "cosmetic rhinoplast*" OR "aesthetic rhinoplast*" OR "elective rhinoplast*" OR "cosmetic septorhinoplast*" OR "aesthetic septorhinoplast*" OR "elective septorhinoplast*" OR "tip plasty" OR "aesthetic rhinoseptoplast*" OR "cosmetic rhinoseptoplast*" OR "elective rhinoseptoplast*" OR "ethnic rhinoplast*" OR "nasal reduct*" OR "nose reduct*" OR "nasal aug*" OR "nose aug*" OR "nasal *shap*" OR "aesthetic nose surg*" OR "cosmetic nasal surg*" OR "aesthetic nasal surg*" OR "blepharoplast*" OR "cosmetic* blepharoplast*" OR "aesthetic* blepharoplast*" OR "elective blepharoplast*" OR "cosmetic eye* surg*" OR "aesthetic eye* surg*" OR "elective eye* surg*" OR "cosmetic oculoplast*" OR "aesthetic oculoplast*" OR "elective oculoplast*" OR "double eyelid* surg*" OR "canthopex*" OR "canthal susp*" OR "epicanthoplast*" OR "*eye* rejuv*" OR "browplast*" OR "*brow* lift*" OR "*brow* susp*" OR "*brow* surg*" OR "browpex*" OR "rhytidectom*" OR "rhytidoplast*" OR "facial lift*" OR "face lift*" OR "facelift*" OR "facial rejuv*" OR "forehead lift*" OR "temporoplast*" OR "temporal lift*" OR "cheek lift*" OR "malar lift*" OR "neck lift*" OR "neck rejuv*" OR "plastysmaplast*" OR "cervicoplast*" OR "surg* fac* rejuv*" OR "surg* neck* rejuv*" OR "cosmetic *fac* surg*" OR "aesthetic *fac* surg*" OR "elective fac* surg*" OR "aesthetic neck surg*" OR "lip lift*" OR "corner lift*" OR "lip surg*" OR "surg* lip* aug*" OR "augm* lip" OR "lip reduct*" OR "aesthetic ear* surg*" OR "cosmetic ear* surg*" OR "aesthetic otoplast*" OR "cosmetic otoplast*" OR "ear* rejuv*" OR "pinnaplast*" OR "ear pinning" OR "ear reduct*" OR "mentoplast*" OR "aesthetic chin surg*" OR "cosmetic chin surg*" OR "aesthetic genioplast*" OR "cosmetic genioplast*" OR "chin *shap*" OR "chin augment*" OR "chin reduct*" OR "jaw *shap*" OR "jaw augment*" OR "jaw reduct*" OR "*face reshap*" OR "*facial reshap*" OR "*facial *shap* surg*" OR "*face *shap* surg*" OR "facial implant*" OR "bichectom*" OR "profiloplast*" OR "profileplast*" OR "*cheek *shap*" OR "malar *shap*" OR "cheek aug*" OR "malar aug*" OR "malarplast*" OR "*malar plast*" OR "*facial *suction*" OR "*face *suction*" OR "*neck liposuct*" OR "neck contour*" OR "*facial implant*" OR "*face implant*" OR "cosmetic facial implant*" OR "aesthetic facial implant*" OR "temporal implant*" OR "frontal implant*" OR "lip implant*" OR "nose implant*" OR "nasal implant*" OR "jaw* implant*" OR "chin implant*" OR "mandib* angle* implant*" OR "malar implant*" OR "*cheek* implant*" OR "zygoma* implant*" OR "rhinoplast*"OR "otoplast*" OR "facial asymmetr*" OR "face asymmetr*" OR "elective *fac* surg*" OR "*jaw surg*" (Topic) |
|  | | |
| **#4** | **(#2 OR #3)**  **n= 65.836** | "cosmetic breast implant*" OR "aesthetic breast implant*" OR "elective breast implant*" OR "cosmetic breast surg*" OR "aesthetic breast surg*" OR "elective breast surg*" OR "elective breast aug*" OR "cosmetic breast aug*" OR "aesthetic breast aug*" OR "breast lift*" OR "breast* symmetrization*" OR "cosmetic mastopex*" OR "aesthetic mastopex*" OR "elective mastopex*" OR "aesthetic breast reduct*" OR "cosmetic breast reduct*" OR "elective breast reduct*" OR "mammaplast*" OR "mammoplast*" OR "abdominoplast*" OR "cosmetic abdominoplast*" OR "aesthetic abdominoplast*" OR "elective abdominoplast*" OR "tumm* tuck*" OR "panniculectom*" OR "lipoabdominoplast*" OR "momm* makeover*" OR "body contour*" OR "fat suction*" OR "abdominal etching" OR “liposuction*” OR "cosmetic liposuct*" OR "aesthetic liposuct*" OR "elective liposuct*" OR "aesthetic contour*" OR “body contour* surg*” OR “surg* body contour*” OR “lipectom*” OR “liposculptur*” OR "lipoplast*" OR "lift* surg*" OR "mons susp*" OR "mons pubis susp*" OR "mons lipo*" OR "mons pubis lipo*" OR "mons pubis reduct*" OR "cosmetic monsplast*" OR "pubic lift*" OR "pubic contour*" OR "limb contour*" OR "extremit* contour*" OR “gluteal augmentation*”OR "gluteal implant*" OR “gluteoplast*” OR “Brazilian butt lift” OR "gluteal reduct*" OR "gluteal lift*" OR "buttock* implant*" OR “brachioplast*” OR "arm* contour*" OR "upper limb* contour*" OR "cosmetic arm*" OR "aesthetic arm*" OR “upper arm lift*” OR "biceps* implant*" OR "deltoid implant*" OR “thighplast*" OR "aesthetic lower limb*" OR "cosmetic lower limb*" OR "thigh lift*" OR "thigh lipo*” OR "aesthetic calf" OR "cosmetic calf" OR "calf implant*" OR "calf reduct*" OR "calf aug*" OR "leg contour*" OR "extremit* contour*" OR "lower limb* contour*" OR "aesthetic leg*" OR "cosmetic leg*" OR "cosmetic implant*" OR "aesthetic implant*" OR "aesthetic body implant*" OR "aesthetic genit* surg*" OR "cosmetic genit* surg*" OR "genital cosmetic surg*" OR "genital aesthetic surg*" OR "genit* rejuv*" OR "cosmetic gynecology" OR "vulvovaginal aesthetic surg*" OR "aesthetic labiaplast*" OR "cosmetic labiaplast*" OR "cosmetic labioplast*" OR "aesthetic labioplast*" OR "aesthetic labia minora reduct*" OR "cosmetic labia minora reduct*" OR "aesthetic labial reduct*" OR "cosmetic labial reduct*" OR "labia majora reduct*" OR "majoraplast*" OR "labia majora aug*" OR "gynecomastia surg*" OR "pectoral implant" OR "scrot* rejuv*" OR "cosmetic urolog*" OR "aesthetic scrotoplast*" OR "cosmetic phalloplast*" OR "aesthetic phalloplast*" OR "cosmetic scrotoplast*" OR "aesthetic scrotoplast*" OR "cosmetic penoplast*" OR "aesthetic penoplast*" OR "scrot* lift*" OR "cosmetic* surg*" OR "aesthetic* surg*" OR "esthetic* surg*" OR "cosmetic* plastic surg*" OR "aesthetic* plastic surg*" OR "esthetic* plastic surg*" OR "elective plastic surg*" OR "breast* implant*" OR "breast* reduct*" OR "breast* aug*" OR "mastopex*" (Topic) or "hair transplant*" OR "follicular unit transplant*" OR "surgical hair restoration*" OR "hair* implant*" OR "cosmetic rhinoplast*" OR "aesthetic rhinoplast*" OR "elective rhinoplast*" OR "cosmetic septorhinoplast*" OR "aesthetic septorhinoplast*" OR "elective septorhinoplast*" OR "tip plasty" OR "aesthetic rhinoseptoplast*" OR "cosmetic rhinoseptoplast*" OR "elective rhinoseptoplast*" OR "ethnic rhinoplast*" OR "nasal reduct*" OR "nose reduct*" OR "nasal aug*" OR "nose aug*" OR "nasal *shap*" OR "aesthetic nose surg*" OR "cosmetic nasal surg*" OR "aesthetic nasal surg*" OR "blepharoplast*" OR "cosmetic* blepharoplast*" OR "aesthetic* blepharoplast*" OR "elective blepharoplast*" OR "cosmetic eye* surg*" OR "aesthetic eye* surg*" OR "elective eye* surg*" OR "cosmetic oculoplast*" OR "aesthetic oculoplast*" OR "elective oculoplast*" OR "double eyelid* surg*" OR "canthopex*" OR "canthal susp*" OR "epicanthoplast*" OR "*eye* rejuv*" OR "browplast*" OR "*brow* lift*" OR "*brow* susp*" OR "*brow* surg*" OR "browpex*" OR "rhytidectom*" OR "rhytidoplast*" OR "facial lift*" OR "face lift*" OR "facelift*" OR "facial rejuv*" OR "forehead lift*" OR "temporoplast*" OR "temporal lift*" OR "cheek lift*" OR "malar lift*" OR "neck lift*" OR "neck rejuv*" OR "plastysmaplast*" OR "cervicoplast*" OR "surg* fac* rejuv*" OR "surg* neck* rejuv*" OR "cosmetic *fac* surg*" OR "aesthetic *fac* surg*" OR "elective fac* surg*" OR "aesthetic neck surg*" OR "lip lift*" OR "corner lift*" OR "lip surg*" OR "surg* lip* aug*" OR "augm* lip" OR "lip reduct*" OR "aesthetic ear* surg*" OR "cosmetic ear* surg*" OR "aesthetic otoplast*" OR "cosmetic otoplast*" OR "ear* rejuv*" OR "pinnaplast*" OR "ear pinning" OR "ear reduct*" OR "mentoplast*" OR "aesthetic chin surg*" OR "cosmetic chin surg*" OR "aesthetic genioplast*" OR "cosmetic genioplast*" OR "chin *shap*" OR "chin augment*" OR "chin reduct*" OR "jaw *shap*" OR "jaw augment*" OR "jaw reduct*" OR "*face reshap*" OR "*facial reshap*" OR "*facial *shap* surg*" OR "*face *shap* surg*" OR "facial implant*" OR "bichectom*" OR "profiloplast*" OR "profileplast*" OR "*cheek *shap*" OR "malar *shap*" OR "cheek aug*" OR "malar aug*" OR "malarplast*" OR "*malar plast*" OR "*facial *suction*" OR "*face *suction*" OR "*neck liposuct*" OR "neck contour*" OR "*facial implant*" OR "*face implant*" OR "cosmetic facial implant*" OR "aesthetic facial implant*" OR "temporal implant*" OR "frontal implant*" OR "lip implant*" OR "nose implant*" OR "nasal implant*" OR "jaw* implant*" OR "chin implant*" OR "mandib* angle* implant*" OR "malar implant*" OR "*cheek* implant*" OR "zygoma* implant*" OR "rhinoplast*"OR "otoplast*" OR "facial asymmetr*" OR "face asymmetr*" OR "elective *fac* surg*" OR "*jaw surg*" (Topic) |
|  | | |
| **#5** | **( #1 AND #4)**  **n= 1.210** | |
