# Supplementary material for: The Impact of Artificial Intelligence on Facial Aesthetic Surgery: A Systematic Review
Source: Aesthet Surg J Open Forum. 2026 Jun 23;8:ojag114. doi: 10.1093/asjof/ojag114 (PMC13345743; doi:10.1093/asjof/ojag114)
Supplement: ojag114_Supplementary_Data [file ojag114_supplementary_data.zip › Supplementary Table 1.docx]

**Table 1A. Main characteristics of included studies - Lip lift surgery**

| **Author(s),**  **Year** | Huang et al.,  2025 |
| --- | --- |
| **Country** | Multicenter |
| **Sample size**  **(patients)** | 4 |
| **Type of surgery** | Lip lift |
| **AI application** | Simulation of postoperative results |
| **AI model(s)/**  **algorithm(s)** | DALL-E2 |
| **AI technique,**  **architecture** | Deep learning,  Transformer |
| **AI sourcing** | Commercially-available |
| **Stage of care** | Treatment planning |
| **Study design** | Feasibility |
| **Comparator group** | - |
| **Outcome** | 1. Realism of AI-generated images  2. Nose-lip distance |
| **Outcome measure** | 1. Accuracy  2. Milimeters |
| **Main findings** | In a case series, AI was used to generate visual representations of lip-lift outcomes. Of the prompts tested, "normal-appearing lips lifted upward" produced the most realistic images. The AI’s predicted under- and overcorrections were subtle, about 1.2 mm of modifications, which may not be achievable in real practice. |
| **AI impact** | **-** |

**Table 1B. Main characteristics of included studies - Brow lift surgery**

| **Author(s),**  **Year** | Boonipat et al.,  2021 | Zhu et al.,  2023 | Hebel et al.,  2025 |
| --- | --- | --- | --- |
| **Country** | USA | USA | Multicenter |
| **Sample size**  **(patients)** | 52 | 53 | 59 |
| **Type of surgery** | Brow lift | Brow lift | Brow lift |
| **AI application** | 1. Emotion intensity  2. Facial action unit (AUs) intensity | 1. Emotion intensity  2. Facial action unit (AUs) intensity | 1. Emotion intensity  2. Facial action unit (AUs) intensity |
| **AI model(s)/**  **algorithm(s)** | FaceReader, Noldus Information Technology BV, Wageningen, the Netherlands | FaceReader, Noldus Information Technology BV, Wageningen, the Netherlands | FaceReader, Noldus Information Technology BV, Wageningen, the Netherlands |
| **AI technique,**  **architecture** | Deep learning,  CNN | Deep learning,  CNN | Deep learning,  CNN |
| **AI sourcing** | Commercially-available | Commercially-available | Commercially-available |
| **Stage of care** | Outcome assessment | Treatment planning | Outcome assessment |
| **Study design** | Feasibility | Feasibility | Feasibility |
| **Comparator group** | - | **-** | **-** |
| **Outcome** | 1. Emotions  2. Facial action units  3. Brow elevation | 1.Emotions  2. Facial action units (AUs) | 1.Emotions  2. Facial action units (AUs) |
| **Outcome measure** | 1.Intensity  2. Intensity  3.Milimeters | 1.Intensity  2. Intensity | 1.Intensity  2. Intensity |
| **Main findings** | AI-based before-and-after analyses of facial emotion and action unit intensity were conducted in brow lift patients. Postoperatively, the AI detected significantly less anger and more happiness (p < 0.05), with no significant changes in sadness, fear, or surprise. Action unit analysis showed a decrease in brow-lowering and an increase in brow-raising units after surgery. | The authors used AI to study how brow rotation affects facial emotion and action unit intensity in brow-lift patients. Only larger brow rotations produced significant changes in emotional expression, mainly in sadness and neutrality. In the AI-based action unit analysis, only larger brow rotations significantly changed intensity, with outer brow raiser units increasing as positive rotation increased, consistent with the primary emotion findings. | In a retrospective analysis, AI was used to compare two brow lift techniques. Facial emotion and action unit analyses found significant emotional improvements -more happiness and less anger- only in the standard brow lift group. Both groups showed significant decreases in AI‑derived brow‑lowering units, paralleling manually measured brow elevation. |
| **AI**  **impact** | **-** | **-** | **-** |

CNN, Convolutional Neural Network;

**Table 1C. Main characteristics of included studies - Facial bony contour surgery**

| **Author(s),**  **Year** | Yan et al.,  2023 | Park et al.,  2024 | Qiu et al.,  2025 |
| --- | --- | --- | --- |
| **Country** | China | Korea | China |
| **Sample size**  **(patients)** | 50 | 72 | 6 |
| **Type of surgery** | Mandibular angle ostectomy | Zygoma surgery | Zygoma surgery |
| **AI model(s)/**  **algorithm(s)** | N/A | Open-source:  1.MediaPipe Face Mesh  2. Zero-reference DeepCurve Estimation (Zero-DCE)  3. Hessian filtering | N/A |
| **AI technique,**  **architecture** | Deep learning,  Hybrid | Deep learning,  Hybrid | Deep learning,  CNN |
| **AI application** | Design standardized mandibular  angle ostectomy guide plates | Analyze cheek sagging | Zygomatic osteotomy design system |
| **AI sourcing** | Proprietary | Proprietary | Proprietary |
| **Stage of care** | Treatment planning | Outcome assessment | Treatment planning |
| **Study design** | Comparative | Feasibility | Comparative |
| **Comparator group** | Manual design by surgeons | - | Manually designed (surgeon's) osteotomy plans |
| **Outcome** | 1. Symmetry  2. Safety  3. Design time  4. Shape matching | 1. Facial sagging  2. Cheek curvature changes  3. Nasolabial fold depth  4. Marionette line depth | 1. Symmetry  2. Safety.  3. Effectiveness  4. Aesthetic outcomes |
| **Outcome measure** | 1. Bilateral difference in mandibular ramus height in milimeters;  2. Mean nerve distance (milimeters) + Safety rate (% safe designs)  3. Seconds  4. Shape matching: Mandibular angle + Bigonial /Bizygomatic distance ratio. | 1. Y-values of 12 cheek landmarks  2. Ratio = curved area/lower cheek  3. Numerical value (z-value)  4. Numerical value. (z-value) | 1.Symmetry indeces; 2.Distance osteotomy surface-infraorbital nerve (mm.) + Safety compliance rates; 3.Shape + location compliance  4. ANA scores |
| **Main findings** | Artificial intelligence was evaluated against resident surgeons for designing mandibular angle ostectomy guide plates. Compared with manual designs, AI-generated plates achieved a greater safety distance (p < 0.001), a higher safety rate (96% vs. 52%, p < 0.001), and required less design time, with no significant differences in surgical symmetry, plate shape, match accuracy, or aesthetic angle ratios. | AI-based image analysis was used to determine whether cheek sagging occurs after zygoma reduction surgery. Pre- and post-surgery AI-assessments showed no significant changes in facial sagging indices, cheek curvature, nasolabial folds, or marionette lines, confirming that no cheek sagging occurred after surgery. | AI-driven zygomatic osteotomy design system was compared against surgeon's manual designs. AI-generated designs showed non-inferior safety (100% vs. 98.75% compliance) and consistently larger safety margins to neural structures. The AI system also achieved significantly higher bilateral symmetry than manual designs (p < 0.05) and improved aesthetic outcomes. |
| **AI**  **impact** | 1.Symmetry: Non-inferior  2.Safety: Superior  3.Design time: Superior  4. Shape matching: Non-inferior | - | 1.Safety: Non-inferior  2. Symmetry: Non-inferior  3. Effectiveness: Non-inferior  4. Aesthetics: Superior |

N/A, not applicable; CNN, Convolutional Neural Network;

**Table 1D. Main characteristics of included studies - Hair Transplant**

| **Author(s),**  **Year** | Erdogan et al.,  2020 | Hwang et al.,  2021 | Zhu et al.,  2024 |
| --- | --- | --- | --- |
| **Country** | Turkey | Korea | China |
| **Sample size**  **(patients)** | 47 | 374 pre-op images;  168 post-op images | 13 |
| **Type of surgery** | Hair transplant | Hair transplant | Hair transplant |
| **AI application** | Follicle counting;  Hair thicknessmeasurement;  Calculates donor capacity and coverage value;  Extracted and placed graft counting;  Scar trace detection;  Calculates transection rates | Surgical results simulation | 1. Image-based follicular unit recognition (analyzes the angle, depth, orientation, and density for each hair follicle)  2. Automated targeting and robotic needle alignment |
| **AI model(s)/**  **algorithm(s)** | KEBOT Robotic System:  1. RetinaNet–ResNet101 backbone  2. SegNet–VGG16 backbone | 1. CycleGAN;  2. U-Net;  3. LinkNet;  4. Feature Pyramid Network (with ResNet-34 encoders) | ARTAS Robotic System |
| **AI technique,**  **Architecture** | Deep learning,  CNN | Deep learning,  GANs | Machine learning,  N/A |
| **AI sourcing** | Hybrid | Hybrid | Proprietary |
| **Stage of care** | Outcome assessment;  Patient assessment | Treatment planning | Intervention;  Treatment planning |
| **Study design** | Technical Development;  Technical Validation | Technical Development;  Technical Validation | Comparative |
| **Comparator group** | 1. Follicular unit count, Hair density, Total hair count, graft counts: manual nurse labeling (ground truth);    2. Hair thickness: Scanning electron microscopy | Other other GAN-based  image translation methods | Manual follicular unit extraction |
| **Outcome** | Pre-operative:  1. Follicular unit detection  2. Hair density  3. Hair Thickness  4. Coverage Value      Post-operative:  1. Transection rates | 1. Structural image preservation ;  2. Region of interest detection accuracy  3.Post-op and predictive image similariry | 1. Technical efficiency;  2. Safety;  3. Patient Satisfaction |
| **Outcome measure** | Pre-operative:  1. Follicular units /cm²  2. Average hairs per follicle  3. Diameter  4. Follicular units/cm² × density × diameter    Post-operative:  1. Extracted grafts count - placed grafts count. | 1. Structural Similarity Index Measure (SSIM);  2. Intersection over Union (IoU);  3. FiD (Frechet Inception Distance); | 1. Yield rates, discard rates, transection rates  2. Pain, infection, scarring  3. Follow-up feedback |
| **Main findings** | An AI-driven algorithm was developed and evaluated for both pre- and post-operative phases of hair implant surgery. Pre-operatively, it achieved near-perfect follicular unit detection with minimal density misclassification, keeping errors in hair count, shaft thickness, and coverage value index calculations below 5%. Post-operatively, it kept errors in extracted and implanted graft counts and transection rates under 5% and accurately traced scars. | AI was used to generate predicted post–hair transplant images from preoperative photographs, and compared its performance with existing methods. Image quality metrics showed a statistically significant improvement (p < 0.02). The Fréchet Inception Distance indicated greater image realism up to λ = 10 (p < 0.05). Overall, the model achieved superior region-of-interest detection compared with baseline models. | In a randomized split-scalp study, an AI-powered hair-implant robot (ARTAS) was compared with manual follicular unit extraction (FUE). Yield and transection rates were similar between methods (p > 0.05). ARTAS performed better for single-hair follicular units (p < 0.05), whereas manual extraction had lower discard rates. Overall, the AI system was non-inferior, with both methods showing comparable safety, infection, pain and satisfaction profiles. |
| **AI**  **impact** | 1. Follicular unit count: non-inferior  2. Calculated density: inferior  3. Total hair count: inferior  3. Graft counting: non-inferior  4. Hair thickness: non-inferior | Image ROI (region of interest) detection: superior | 1. Safety: non-inferior  2.Satisfaction rates: non-inferior |

N/A, not applicable; CNN, Convolutional Neural Network; GAN, Generative Adversial Network;

**Table 1E. Main characteristics of included studies - Blepharoplasty**

| **Author(s),**  **Year** | Şimşek et al.,  2021 | Qu et al.,  2022 | Song et al.,  2023 | Chiou et al.,  2024 | Kreh et al.,  2025 | Lian et al.,  2025 |
| --- | --- | --- | --- | --- | --- | --- |
| **Country** | Turkey | China | Korea | Taiwan | USA | China |
| **Sample size**  **(patients)** | 55 | 64 | 77 | 150 | 153 | 454 |
| **Type of surgery** | Blepharoplasty | Blepharoplasty | Blepharoplasty | Blepharoplasty | Blepharoplasty | Blepharoplasty |
| **AI application** | Facial landmark detection;  Automated measurement of: palpebral distance, eye-opening area, and average eyebrow height for both eyes | 3D modeling;  Calculates pouch volume, wrinkle depth, and tissue contour metrics for surgical planning; Suggests incision symmetry and resection zones | MRD1 (Marginal reflex distance 1) measurement | Age estimation | Age estimation | Early-aging identification;  Aesthetic procedure reccomendations |
| **AI model(s)/**  **algorithm(s)** | DLIB-ML Toolkit | N/A | RITnet | 1. Amazon Rekognition (Seattle, WA)  2.Microsoft Azure Face (Redmond, WA)  3. Face++ Detect (Beijing, China)  4. IfErdo face detection (New York, NY) | 1.Face++ (Megvi, Beijing, China) 2.Betaface (Munich, Germany)  3.Facelytics (Wassa, France)  4.Everypixel (Everypixel Media Innovation Group, Singapore). | HATrans |
| **AI technique,**  **architecture** | Machine learning,  Classical (regression) | Deep learning,  CNN | Deep learning,  CNN | Deep learning,  CNN | Deep learning,  CNN | Deep learning,  Transformer |
| **AI sourcing** | Open-source | Proprietary | Open-source | Commercially-available | Commercially-available | Proprietary |
| **Stage of care** | Outcome assessment | Treatment planning | Patient assessment | Outcome assessment | Outcome assessment | Outcome assessment |
| **Study design** | Feasibility | Comparative | Comparative | Feasibility | Feasibility | Feasibility |
| **Comparator group** | - | Doctor's experience alone | 1. Manual method  2. Image-based, computerized methods | - | - | - |
| **Outcome** | Periocular morphometrics | 1. Reconstruction effect 2. Surgical effect  3. Aesthetic scores 4.Complications | Marginal reflex distance 1 | 1. Age Detection Accuracy  2. Estimated age reduction  2. Consistency, Reproducibility  3. Clinical: sex differences, type of surgery differences | 1. Age estimation accuracy score  2. Clinical: Percieved age reduction | 1. Model performance metrics |
| **Outcome measure** | 1.Palpebral distance  2. Average eyebrow height  3. Eye opening area | 1. Similarity (%) + Efficiency (seconds)  2. Pouch degree (grade 1-3), Lower eyelid wrinkles (Fitzpatrick grading). Eyelid lacrimal sulculs filling, Skin gloss (gloss meter)  3. Composite aesthetic score (5 items)  4. Eversion, retraction, hematoma event reporting | 1. Manual measurements  2. ImageJ software analysis of Nikon D7500 digital camera images  3. ImageJ software analysis of infrared camera images  4. Novel, deep learning method with infrared camera image | 1. Intraclass Correlation Coefficient (ICC), Mean Absolute Error (MAE)  2. Post-op image estimated age - Pre-op image estimated age  3. Descriptive data | 1. Score = average true preoperative age/average estimated preoperative age ×100  2. Statistical testing for variables.... | 1. Sensitivity, Spoecificity, Accuracy, Area Under the ROC Curve (AUC), Negative Likelihood Ratio (NLR), Negative Predictive Value (NPV), Positive Likelihood Ratio (PLR), Positive Predictive Value (PPV), Harmonic mean of Precision and Recall (F1 scoare) |
| **Main findings** | In two blepharoplasty groups, AI was used to evaluate changes in palpebral distance, eye-opening area, and average eyebrow height. The analysis showed that combined blepharoplasty and Müller muscle resection resulted in greater eyelid opening (p < 0.04) and more pronounced eyebrow descent than blepharoplasty alone (p = 0.01). The AI method provided a standardized and objective tool for outcome assessment. | A comparative study evaluated the effect of incorporating AI into surgeons’ experience for blepharoplasty surgical planning. With a 3D CNN-based eyelid model, surgeons achieved greater reductions in eye bag severity, wrinkle depth, and tear-trough deformity, higher aesthetic ratings, and a lower complication rate (from 28% to 13%). | Four MRD1 datasets from blepharoplasty patients were collected using three measurement methods. AI-based MRD1 measurements showed no significant mean difference compared with manual or computer-assisted methods. The AI method matched infrared measurements most closely, with the highest correlation (r = 0.822), the smallest mean difference, the narrowest limits of agreement, and similar value distribution; this suggests that the AI-based method outperforms traditional manual techniques. | Four AI-based age-estimation models were used to evaluate the anti-aging effects of blepharoplasty. Face++ was the most accurate, followed by the mean prediction across models. AI-based age estimates were stable over time. AI assessments showed a statistically significant rejuvenation effect of blepharoplasty (p < 0.0001), with a mean apparent age reduction of −1.68 ± 4.03 years, especially in men and in those undergoing combined blepharoplasty. | AI analysis of before-and-after age assessments in patients undergoing periorbital cosmetic surgery showed that Face++ was the most accurate of four CNN models, while the others tended to underestimate true age. Periorbital rejuvenation reduced perceived age by an average of 1.03 years (p < .001), and brow lifts produced an additional independent reduction of 1.43 years (p = 0.031). | The AI model detected signs of periorbital aging and offered accessible, timely treatment suggestions through a phone-based app. Surgeons widely accepted the AI model's recommendations, with indirect acceptance rates between 89.5% and 94%. |
| **AI**  **impact** | - | 1. Aesthetic rating: superior  2. Safety: superior  3. Surgical outcomes: superior | 1. Superior: to manual method  2. Non-inferior: to infrared methods | - | - | Accuracy: superior  Sensitivity: superior  Specificity: superior |

N/A, not applicable; CNN, Convolutional Neural Network;

**Table 1F. Main characteristics of included studies - Surgical rhinoplasty**

| **Author(s),**  **Year** | Dorfman et al.,  2019 | Khetpal et al.,  2022 | Jafargholkhanloo et al.,  2023 | Li et al.,  2023 | Suh et al.,  2024 | Yalçın et al.,  2025 |
| --- | --- | --- | --- | --- | --- | --- |
| **Country** | USA | USA | Iran | China | Korea | Turkey |
| **Sample size**  **(patients)** | 100 | 124 | 100 | 209 | 4 | 244 |
| **Type of surgery** | Rhinoplasty | Rhinoplasty | Rhinoplasty | Rhinoplasty | Rhinoplasty | Septorhinoplasty |
| **AI application** | Age estimation | 1. Age estimation  2. Facial attractiveness analysis | Facial Landmark Localization | Ideal nose simulation | Development of customized nasal implants | Age estimation |
| **AI model(s)/**  **algorithm(s)** | Microsoft Azure Face API (Redmond, WA) | Haystack AI, (New York, NY) | 1. Fuzzy C-means with Grey Wolf Optimization;  2. Cascade regression model;  3. Local Phase Quantization | FoldingNet | 1. U-net  2. Snake | ''AgeBot: How Old am I? App." |
| **AI technique,**  **Architecture** | Machine learning,  CNN | Machine learning,  CNN | Machine learning,  Classical | Deep learning,  DNN | Machine learning,  Hybrid | Deep learning,  CNN |
| **AI sourcing** | Commercially-available | Commercially-available | Proprietary | Proprietary | Hybrid | Commercially-available |
| **Stage of care** | Outcome assessment | Outcome assessment | Patient assessment;  Outcome assessment | Treatment planning | Treatment planning | Outcome assessment |
| **Study design** | Feasibility | Feasibility | Technical validation study,  Comparative | Technical development,  Technical validation study | Feasibility | Feasibility |
| **Comparator group** | - | - | Manual measurements | - | - | - |
| **Outcome** | Age acurracy | 1. Apparent age  2. Attractiveness | 1. Angular measurements | 1.Performance;  2.Reconstruction accuracy;  3.Design Time | 1. Error | 1. Age estimation  2. Patient satisfaction |
| **Outcome measure** | Correlation coefficient | 1. Years  2. Facial attrcativeness scores (1-10) | 1. Average values of: nasofrontal, nose tip, nasolabial, mentolabial, nasomental, facial convexity including nose, facial convexity excluding nose, projection of the upper lip to chin angles | 1. Loss Function Convergence (epochs);  2. Euclidean distace  3. Descriptive | 1. Millimeters | 1. Years  2. Satisfaction scores (FACE-Q) |
| **Main findings** | An age-estimation AI application was used on the same patient group before and after rhinoplasty. Actual and predicted preoperative age were strongly correlated (r = 0.91). The CNN algorithm slightly overestimated preoperative age. After open rhinoplasty, patients appeared 3 years younger - a statistically significant anti-aging effect. | AI was used to assess facial age and attractiveness before and after rhinoplasty. Before surgery, patients were estimated to look 2.56 years older than their chronological age, compared with 1.53 years older after surgery; this reflects a significant 1.03-year reduction. AI-based facial attractiveness scores also increased significantly after surgery (p = 0.030). | In a before-and-after rhinoplasty cohort, nine facial angular metrics were measured manually and with an AI-based cascade regression method. Accuracy analysis showed no significant differences between AI-based and clinicians’ manual measurements. The AI method also delivered faster results, and reduced the likelihood of measurement errors. | This study compared rhinoplasty outcomes derived from AI‑generated ideal nose simulations with those based on surgeons’ manual designs. By 900 epochs, the mean Euclidean difference from manual designs was ≤ 0.8 mm. It identified key facial subunits, about 1,000 complex features, and modeled each nasal side independently. The model may provide more natural‑looking results, and nearly real time simulations. | In a case series study, AI supported nasal implant treatment planning by automating image segmentation, estimating nasal cartilage, and designing custom implant shapes. Using extensive data-driven AI methods, the model achieved an error of less than 1 mm. Patients reported high satisfaction with the treatment outcomes. | The anti-aging effect of rhinoplasty was evaluated in a before-and-after rhinoplasty patient cohort. Despite a 25.3 ± 8.7-month follow-up period, the absence of a significant difference between pre- and postoperative perceived ages suggests a potential anti-aging effect of rhinoplasty, especially in older patients and women (p = 0.001). |
| **AI**  **Impact** | - | - | Accuracy:  Non-inferior | - | - | - |

CNN, Convolutional Neural Network; DNN, Deep Neural Network;

**Table 1G. Main characteristics of included studies - Facelift surgery**

| **Author(s),**  **Year** | Gibstein et al.,  2020 | Zhang et al.,  2020 | Bouguila et al.,  2021 | Elliott et al.,  2022 | Hebel et al,  2023 | Du et al.,  2024 | Tiryaki et al.,  2024 |
| --- | --- | --- | --- | --- | --- | --- | --- |
| **Country** | USA | USA | Tunisia | USA | USA | China | Multicenter |
| **Sample size**  **(patients)** | 105 | 50 | 37 | 226 | 32 | 48 | 400 |
| **Type of surgery** | Facelift +/- fat grafting | Facelift | Facelift | Facelift +/- ancillary techniques | Facelift | Facelift | Facial lipofilling |
| **AI application** | Age estimation | Age estimation | Age estimation | Age estimation | Facial emotion and action unit intensity analysis | Age estimation | Decision support system |
| **AI model(s)/**  **algorithm(s)** | 1. Face Plus Plus Megvii  2. Amazon Rekognition  3. Microsoft Azure Face  4.IBM | 1.Face Plus Plus Megvii  2. Amazon Rekognition  3.Microsoft Azure Face  4.IBM | Face Plus Plus Megvii (Beijing, China) | FaceX CNN (Bangladesh, India) | FaceReader (Noldus Information, the Netherlands) | MiVOLO | ML.NET |
| **AI technique,**  **architecture** | Deep learning,  CNN | Deep learning,  CNN | Deep learning,  CNN | Deep learning,  CNN | Deep learning,  CNN | Deep learning,  Transformer | Machine learning,  Classical (regression) |
| **AI sourcing** | Commercially-available | Commercially-available | Commercially-available | Commercially-available | Commercially-available | Open-source | Hybrid |
| **Stage of care** | Outcome assessment | Outcome assessment | Outcome assessment | Outcome assessment | Outcome assessment | Outcome assessment | Treatment planning |
| **Study design** | Feasibility | Feasibility | Feasibility | Feasibility | Feasibility | Feasibility | Feasibility |
| **Comparator group** | - | - | - | - | - | 10 blinded naive observers | - |
| **Outcome measure** | 1. Age accuracy score (actual preop age/estimated  neural network preop age × 100)  2. FACE-Q scores | 1.  Age accuracy score (actual preop age/estimated  neural network preop age × 100)  2. FACE-Q scores | 1. Years  2. 4 linear + 3 angular facial metrics | 1. Age accuracy score | 1. Percentage (%)  2. Value (0-5) | 1. Mean absolute error | N/A |
| **Outcome** | 1. Accuracy  2. Patient satisfaction | 1. Accuracy  2. Patient satisfaction | 1. Age  2. Biometric facial features | 1. Accuracy | 1. Emotion intensity  2. Facial action unit intensity | 1. Accuracy | Indirect clinical effectiveness |
| **Main findings** | Four AI models estimated the rejuvenating effects of various facelift techniques with good preoperative age-detection accuracy. Skin-only facelifts produced a smaller AI-estimated age reduction than SMAS plication or SMASectomy. Adding fat grafting increased the mean AI-estimated age reduction by 2.1 years, and higher AI-estimated rejuvenation closely correlated with greater patient satisfaction. | Pre- and postoperative facelift photographs were analyzed using AI-based age estimation. All four neural networks accurately estimated patient age. After surgery, AI-estimated age reduction strongly correlated with FACE-Q satisfaction scores (R² ≈ 0.88–0.92). Notably, patients perceived themselves as significantly younger than the AI estimates, consistently overestimating their rejuvenation (−6.7 vs −4.3 years, p = 0.00158). | AI-based age assessments were conducted before and after surgery in the same facelift patient group. Preoperatively, AI-estimated ages closely matched patients’ chronological ages. Postoperatively, the AI detected a rejuvenation effect of approximately 5.57 years, with the mean estimated age decreasing from 64.54 to 58.97 years. | AI-based age estimation was used in patients treated with different facelift techniques. The model showed 96% accuracy in preoperative age detection, with a tendency to overestimate age. After surgery, AI assessment indicated a significant reduction in estimated age. No single facelift technique or ancillary procedure was superior, but a combined approach more effectively reduced apparent age. | AI facial emotion and action unit intensity analysis was performed in a group of facelift patients. Significant improvements in AI emotion metrics occurred only in the High-SMAS group: happiness rose (p < 0.01), and anger decreased (p = 0.03). Facial action unit analysis showed fewer negative and more positive units, with the magnitude of change varying by technique. | Both AI and human observers estimated age of before-and-after facelift patients,. AI was more precise, with a mean absolute error of 3.34 years (versus 4.82 years for human observers) and a Pearson correlation of 0.90. AI also identified a smaller, but statistically significant degree of rejuvenation. AI estimations of apparent age patterns aligned with the authors’ clinical observations and experience. | The authors developed an AI-driven predictive engine using a multicenter dataset of 3,200 procedures. As a decision support system for facial lipofilling, it guides treatment for patients who fall outside standard golden ratios, enabling personalized care and supporting less experienced practitioners. |
| **AI**  **Impact** | - | - | - | - | - | Age estimation: superior | - |

N/A, not applicable; CNN, Convolutional Neural Network;
