# Supplementary material for: The Impact of Artificial Intelligence on Facial Aesthetic Surgery: A Systematic Review
Source: Aesthet Surg J Open Forum. 2026 Jun 23;8:ojag114. doi: 10.1093/asjof/ojag114 (PMC13345743; doi:10.1093/asjof/ojag114)
Supplement: ojag114_Supplementary_Data [file ojag114_supplementary_data.zip › Supplementary Tables 2-8.docx]

**Table 2. Applications of AI in lip lift surgery**

| **Author(s),**  **Year** | **Study Type** | **Stage of clinical care** | **AI application** | **Main findings** |
| --- | --- | --- | --- | --- |
| Huang et al.,  2025 | Feasibility | Treatment planning | Simulation of postoperative results | In a case series, AI was used to generate visual representations of lip-lift outcomes. Of the prompts tested, "normal-appearing lips lifted upward" produced the most realistic images. The AI’s predicted under- and overcorrections were subtle, about 1.2 mm of modifications, which may not be achievable in real practice. |

**Table 3. Applications of AI in brow lift surgery**

| **Author(s),**  **Year** | **Study Type** | **Stage of clinical care** | **AI application** | **Main findings** |
| --- | --- | --- | --- | --- |
| Boonipat et al., 2021 | Feasibility | Outcome assessment | Facial emotion and action unit intensity analysis | AI-based before-and-after analyses of facial emotion and action unit intensity were conducted in brow lift patients. Postoperatively, the AI detected significantly less anger and more happiness (p < 0.05), with no significant changes in sadness, fear, or surprise. Action unit analysis showed a decrease in brow-lowering and an increase in brow-raising units after surgery. |
| Zhu et al.,  2023 | Feasibility | Treatment planning | Facial emotion and action unit intensity analysis | The authors used AI to study how brow rotation affects facial emotion and action unit intensity in brow-lift patients. Only larger brow rotations produced significant changes in emotional expression, mainly in sadness and neutrality. In the AI-based action unit analysis, only larger brow rotations significantly changed intensity, with outer brow raiser units increasing as positive rotation increased, consistent with the primary emotion findings. |
| Hebel et al.,  2025 | Feasibility | Outcome assessment | Facial emotion and action unit intensity analysis | In a retrospective analysis, AI was used to compare two brow lift techniques. Facial emotion and action unit analyses found significant emotional improvements -more happiness and less anger- only in the standard brow lift group. Both groups showed significant decreases in AI‑derived brow‑lowering units, paralleling manually measured brow elevation. |

**Table 4. Applications of AI in facial bony contour surgery**

| **Author(s),**  **Year** | **Study Type** | **Stage of clinical care** | **AI application** | **Main findings** |
| --- | --- | --- | --- | --- |
| Yan et al.,  2023 | Comparative  (against manual surgeon's design) | Treatment planning | Design of standardized mandibular angle ostectomy guide plates | Artificial intelligence was evaluated against resident surgeons for designing mandibular angle ostectomy guide plates. Compared with manual designs, AI-generated plates achieved a greater safety distance (p < 0.001), a higher safety rate (96% vs. 52%, p < 0.001), and required less design time, with no significant differences in surgical symmetry, plate shape, match accuracy, or aesthetic angle ratios. |
| Park et al.,  2024 | Feasibility | Outcome assessment | Cheek sagging analysis | AI-based image analysis was used to determine whether cheek sagging occurs after zygoma reduction surgery. Pre- and post-surgery AI-assessments showed no significant changes in facial sagging indices, cheek curvature, nasolabial folds, or marionette lines, confirming that no cheek sagging occurred after surgery. |
| Qiu et al.,  2025 | Comparative  (against manual surgeon's design) | Treatment planning | Zygomatic osteotomy design system | AI-driven zygomatic osteotomy design system was compared against surgeon's manual designs. AI-generated designs showed non-inferior safety (100% vs. 98.75% compliance) and consistently larger safety margins to neural structures. The AI system also achieved significantly higher bilateral symmetry than manual designs (p < 0.05) and improved aesthetic outcomes. |

**Table 5. Applications of AI in hair transplant surgery**

| **Author(s),**  **Year** | **Study Type** | **Stage of clinical care** | **AI application** | **Main findings** |
| --- | --- | --- | --- | --- |
| Erdogan et al.,  2020 | Technical Development + Technical Validation; | Patient assessment;  Outcome assessment | Follicle counting;  Hair thickness  measurement;  Donor capacity and coverage value calculus;  Extracted and placed graft counting;  Scar trace detection;  Transection rate calculus | An AI-driven robotic system was developed and evaluated for both pre- and post-operative phases of hair implant surgery. Pre-operatively, it achieved near-perfect follicular unit detection with minimal density misclassification, keeping errors in hair count, shaft thickness, and coverage value index calculations below 5%. Post-operatively, it kept errors in extracted and implanted graft counts and transection rates under 5% and accurately traced scars. |
| Hwang et al., 2021 | Technical Development + Technical Validation | Treatment planning | Surgical results simulation | AI was used for image prediction of hair transplant from preoperative photographs, and compared its performance with existing methods. Performance metrics showed a statistically significant improvement (p < 0.02). The Fréchet Inception Distance indicated greater image realism up to λ = 10 (p < 0.05). Overall, the model achieved superior region-of-interest detection compared with baseline models. |
| Zhu et al.,  2024 | Comparative  (against manual FUE) | Intervention;  Treatment planning | Image-based follicular unit recognition; Automated targeting and robotic needle alignment; Real-time motion control and safety optimization; Records yield, discard and transection rates | In a randomized split-scalp study, an AI-powered hair-implant robot (ARTAS) was compared with manual follicular unit extraction (FUE). Yield and transection rates were similar between methods (p > 0.05). ARTAS performed better for single-hair follicular units (p < 0.05), whereas manual extraction had lower discard rates. Overall, the AI system was non-inferior, with both methods showing comparable safety, infection, pain and satisfaction profiles. |

**Table 6. Applications of AI in blepharoplasty**

| **Author(s),**  **Year** | **Study Type** | **Stage of clinical care** | **AI application** | **Main findings** |
| --- | --- | --- | --- | --- |
| Şimşek et al.,  2021 | Feasibility | Outcome assessment | Facial landmark detection; Automated measurement of: palpebral distance, eye-opening area, and average eyebrow height for both eyes | In two blepharoplasty groups, AI was used to evaluate changes in palpebral distance, eye-opening area, and average eyebrow height. The analysis showed that combined blepharoplasty and Müller muscle resection resulted in greater eyelid opening (p < 0.04) and more pronounced eyebrow descent than blepharoplasty alone (p = 0.01). The AI method provided a standardized and objective tool for outcome assessment. |
| Qu et al.,  2022 | Comparative  (against doctor's experience alone) | Treatment planning | 3D modeling; Calculates pouch volume, wrinkle depth, and tissue contour metrics for surgical planning;  Suggests incision symmetry and resection zones | A comparative study evaluated the effect of incorporating AI into surgeons’ experience for blepharoplasty surgical planning. With a 3D CNN-based eyelid model, surgeons achieved greater reductions in eye bag severity, wrinkle depth, and tear-trough deformity, higher aesthetic ratings, and a lower complication rate (from 28% to 13%). |
| Song et al.,  2023 | Comparative  (against other manual, and image-based computerized methods) | Patient assessment | MRD1 (Marginal reflex distance 1) measurement | Four MRD1 datasets from blepharoplasty patients were collected using three measurement methods. AI-based MRD1 measurements showed no significant mean difference compared with manual or computer-assisted methods. The AI method matched infrared measurements most closely, with the highest correlation (r = 0.822), the smallest mean difference, the narrowest limits of agreement, and similar value distribution; this suggests that the AI-based method outperforms traditional manual techniques. |
| Chiou et al.,  2024 | Feasibility | Outcome assessment | Age estimation | Four AI-based age-estimation models were used to evaluate the anti-aging effects of blepharoplasty. Face++ was the most accurate, followed by the mean prediction across models. AI-based age estimates were stable over time. AI assessments showed a statistically significant rejuvenation effect of blepharoplasty (p < 0.0001), with a mean apparent age reduction of −1.68 ± 4.03 years, especially in men and in those undergoing combined blepharoplasty. |
| Kreh et al.,  2025 | Feasibility | Outcome assessment | Age estimation | AI analysis of before-and-after age assessments in patients undergoing periorbital cosmetic surgery showed that Face++ was the most accurate of four CNN models, while the others tended to underestimate true age. Periorbital rejuvenation reduced perceived age by an average of 1.03 years (p < .001), and brow lifts produced an additional independent reduction of 1.43 years (p = 0.031). |
| Lian et al.,  2025 | Feasibility | Patient assessment | Early-aging identification;  Aesthetic procedure reccomendations | The AI model detected signs of periorbital aging and offered accessible, timely treatment suggestions through a phone-based app. Surgeons widely accepted the AI model's recommendations, with indirect acceptance rates between 89.5% and 94%. |

**Table 7. Applications of AI in rhinoplasty**

| **Author(s),**  **Year** | **Study Type** | **Stage of clinical care** | **AI application** | **Main findings** |
| --- | --- | --- | --- | --- |
| Dorfman et al.,  2019 | Feasibility | Outcome assessment | Age estimation | An age-estimation AI application was used on the same patient group before and after rhinoplasty. Actual and predicted preoperative age were strongly correlated (r = 0.91). The CNN algorithm slightly overestimated preoperative age. After open rhinoplasty, patients appeared 3 years younger - a statistically significant anti-aging effect. |
| Khetpal et al.,  2022 | Feasibility | Outcome assessment | Age estimation  Facial attractiveness analysis | AI was used to assess facial age and attractiveness before and after rhinoplasty. Before surgery, patients were estimated to look 2.56 years older than their chronological age, compared with 1.53 years older after surgery; this reflects a significant 1.03-year reduction. AI-based facial attractiveness scores also increased significantly after surgery (p = 0.030). |
| Jafargholkhanloo et al.,  2023 | Technical validation study,  Comparative | Patient assessment;  Outcome assessment | Facial Landmark Localization | In a before-and-after rhinoplasty cohort, nine facial angular metrics were measured manually and with an AI-based cascade regression method. Accuracy analysis showed no significant differences between AI-based and clinicians’ manual measurements. The AI method also delivered faster results, and reduced the likelihood of measurement errors. |
| Li et al.,  2023 | Technical development + Technical validation study | Treatment planning | Ideal nose simulation | This study compared rhinoplasty outcomes derived from AI‑generated ideal nose simulations with those based on surgeons’ manual designs. By 900 epochs, the mean Euclidean difference from manual designs was ≤ 0.8 mm. It identified key facial subunits, about 1,000 complex features, and modeled each nasal side independently. The model may provide more natural‑looking results, and nearly real time simulations. |
| Suh et al.,  2024 | Technical validation study | Treatment planning | Development of customized nasal implants | In a case series study, AI supported nasal implant treatment planning by automating image segmentation, estimating nasal cartilage, and designing custom implant shapes. Using extensive data-driven AI methods, the model achieved an error of less than 1 mm. Patients reported high satisfaction with the treatment outcomes. |
| Yalçın et al.,  2025 | Feasibility | Outcome assessment | Age estimation | The anti-aging effect of rhinoplasty was evaluated in a before-and-after rhinoplasty patient cohort. Despite a 25.3 ± 8.7-month follow-up period, the absence of a significant difference between pre- and postoperative perceived ages suggests a potential anti-aging effect of rhinoplasty, especially in older patients and women (p = 0.001). |

**Table 8. Applications of AI in facelift/ facial rejuvenation surgery**

| **Author(s),**  **Year** | **Study Type** | **Stage of clinical care** | **AI application** | **Main findings** |
| --- | --- | --- | --- | --- |
| Gibstein et al.,  2020 | Feasibility | Outcome assessment | Age estimation | Four AI models estimated the rejuvenating effects of various facelift techniques with good preoperative age-detection accuracy. Skin-only facelifts produced a smaller AI-estimated age reduction than SMAS plication or SMASectomy. Adding fat grafting increased the mean AI-estimated age reduction by 2.1 years, and higher AI-estimated rejuvenation closely correlated with greater patient satisfaction. |
| Zhang et al.,  2020 | Feasibility | Outcome assessment | Age estimation | Pre- and postoperative facelift photographs were analyzed using AI-based age estimation. All four neural networks accurately estimated patient age. After surgery, AI-estimated age reduction strongly correlated with FACE-Q satisfaction scores (R² ≈ 0.88–0.92). Notably, patients perceived themselves as significantly younger than the AI estimates, consistently overestimating their rejuvenation (−6.7 vs −4.3 years, p = 0.00158). |
| Bouguila et al.,  2021 | Feasibility | Outcome assessment | Age estimation | AI-based age assessments were conducted before and after surgery in the same facelift patient group. Preoperatively, AI-estimated ages closely matched patients’ chronological ages. Postoperatively, the AI detected a rejuvenation effect of approximately 5.57 years, with the mean estimated age decreasing from 64.54 to 58.97 years. |
| Elliott et al.,  2022 | Feasibility | Outcome assessment | Age estimation | AI-based age estimation was used in patients treated with different facelift techniques. The model showed 96% accuracy in preoperative age detection, with a tendency to overestimate age. After surgery, AI assessment indicated a significant reduction in estimated age. No single facelift technique or ancillary procedure was superior, but a combined approach more effectively reduced apparent age. |
| Hebel et al,  2023 | Feasibility | Outcome assessment | Facial emotion and action unit intensity analysis | AI facial emotion and action unit intensity analysis was performed in a group of facelift patients. Significant improvements in AI emotion metrics occurred only in the High-SMAS group: happiness rose (p < 0.01), and anger decreased (p = 0.03). Facial action unit analysis showed fewer negative and more positive units, with the magnitude of change varying by technique. |
| Du et al.,  2024 | Feasibility | Outcome assessment | Age estimation | Both AI and human observers estimated age of before-and-after facelift patients. AI accuracy was superior, with a mean absolute error of 3.34 years (versus 4.82 years for human observers) and a Pearson correlation of 0.90. AI also identified a smaller, but statistically significant degree of rejuvenation. AI estimations of apparent age patterns aligned with the authors’ clinical observations and experience. |
| Tiryaki et al.,  2024 | Feasibility | Treatment planning | Decision spport system (recommends optimal fat volume, graft type, and injection depth) | The authors developed an AI-driven predictive engine using a multicenter dataset of 3,200 procedures. As a decision support system for facial lipofilling, it guides treatment for patients who fall outside standard golden ratios, enabling personalized care and supporting less experienced practitioners. |
